# Limited capacity for rapid acclimation to marine heatwaves in coastal invertebrates

**DOI:** 10.1111/1365-2656.70332

**Published:** 2026-08-16

**Authors:** Mafalda Tomás, Patrice Pottier, Alexandro Autiero, Elena Tamarit‐Castro, Alex A. Berry, Tamzin A. Blewett, Ivan Cadonic, Dustin Doty, Maryane Gradito, Elizabeth C. Hoots, Luis L. Kuchenmüller, Sidney Martin, Vincent Mélançon, Juliane Vigneault, Timothy D. Clark, Zara‐Louise Cowan, Fredrik Jutfelt, Leon Green

**Affiliations:** ^1^ Department of Biological and Environmental Sciences, Faculty of Science and Technology University of Gothenburg Gothenburg Sweden; ^2^ Evolution & Ecology Research Centre, School of Biological, Earth and Environmental Sciences University of New South Wales Sydney New South Wales Australia; ^3^ Division of Ecology and Evolution, Research School of Biology The Australian National University Canberra Australian Capital Territory Australia; ^4^ Department of Marine Sciences, Faculty of Science and Technology University of Gothenburg Gothenburg Sweden; ^5^ Department of Earth Sciences, Faculty of Science and Technology University of Gothenburg Gothenburg Sweden; ^6^ School of Life and Environmental Sciences Deakin University Geelong Victoria Australia; ^7^ Department of Biological Sciences, Faculty of Science University of Alberta Edmonton Canada; ^8^ Groupe de recherche interuniversitaire en limnologie (GRIL), Département de sciences biologiques, Université de Montréal Montréal Québec Canada; ^9^ Département de Biologie, Chimie et géographie Université du Québec à Rimouski Rimouski Canada; ^10^ Maritime Museum & Aquarium Gothenburg Sweden

**Keywords:** acclimation response ratio, biogeographic patterns, climate change, ectotherms, sea surface temperature, thermal plasticity

## Abstract

1. Marine heatwaves are increasing in frequency and intensity worldwide and represent a growing threat to coastal ecosystems and the animals inhabiting them.
2. The vulnerability of marine species to these events depends on several factors, including upper thermal tolerance, the thermal sensitivity of physiological performance, and the capacity for rapid physiological acclimation.
3. We examined upper thermal tolerance and short‐term acclimation capacity in 10 mobile macroinvertebrate species inhabiting shallow coastal ecosystems in the Northeast Atlantic.
4. Individuals were exposed either to ambient thermal conditions at 15°C or to a simulated short‐term marine heatwave of 5 days at 5°C above ambient temperature. We then measured critical thermal maxima (CT_max_) to quantify thermal limits and acclimation responses.
5. Across species, CT_max_ increased significantly following heatwave exposure, showing that these coastal invertebrates can undergo rapid physiological adjustment.
6. However, the magnitude of the acclimation response was modest, indicating that short‐term plasticity provides only limited protection against acute thermal extremes.
7. These limited acclimation responses suggest that some coastal invertebrates may remain vulnerable to intensifying marine heatwaves, particularly taxa with narrow thermal safety margins.
8. Our findings provide an ecologically relevant assessment of thermal performance in coastal invertebrates and contribute to predictions of how nearshore communities may respond to accelerating ocean warming.

## Graphical abstract

Across 10 coastal invertebrate species, we provide a rare multi‐species test of rapid acclimation to realistic marine heatwaves. Responses are consistently weak, revealing that short‐term plasticity cannot keep pace with warming and may lead to underestimated vulnerability in coastal ecosystems.

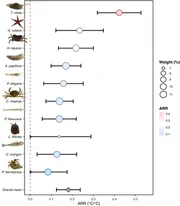

## 1 INTRODUCTION

Oceans are warming at an unprecedented rate, accompanied by an increase in the frequency and intensity of extreme thermal events (IPCC, 2023; Jutfelt et al., 2024). Among the most impactful are marine heatwaves: sustained periods of at least 5 days in which sea temperatures exceed a seasonally varying 90th‐percentile threshold, often severely disrupting marine ecosystems (Hobday et al., 2016; Wernberg et al., 2024). The ecological repercussions can be profound, driving large‐scale mortality, degrading habitats and reshaping species distributions (Oliver et al., 2018; Smith et al., 2023). Marine ecosystems are especially vulnerable because most inhabitants are ectothermic (Willmer et al., 2005), with body temperatures and physiological processes directly regulated by the surrounding environment. Rapid and extreme ocean warming can therefore push these organisms beyond their physiological limits, impairing essential functions and ultimately compromising population viability (De Bonville et al., 2025; Jutfelt et al., 2024).

An organism's vulnerability to heat stress depends on how physiological performance responds to warming and on thermal plasticity, the capacity to make reversible physiological, behavioural or morphological adjustments to temperature change (Jutfelt et al., 2024; Somero, 2010). Thermal acclimation, defined here as shifts in heat tolerance in response to chronic changes in thermal conditions, has been observed across a wide range of species (Gunderson & Stillman, 2015; Morley et al., 2019). Warm acclimation generally raises the acute thermal tolerance often assessed as critical thermal maximum (CT_max_). CT_max_ is commonly estimated using acute thermal ramping assays and represents the upper temperature threshold at which an organism loses motor control, thereby providing a measure of its capacity to withstand acute thermal extremes (De Bonville et al., 2025; Jørgensen et al., 2019; Morgan et al., 2020). Yet, for species already existing at the edge of habitable temperatures, such as those in tropical seas or the upper intertidal, the scope for further adjustment can be minimal (Parry‐Wilson et al., 2024). In these cases, even slight increases in temperature can breach their thermal safety margins, the difference between their upper thermal limits and maximum environmental temperatures, leaving them acutely vulnerable to further warming (Ravaux et al., 2016).

This capacity for adjustment is commonly quantified using the Acclimation Response Ratio (ARR), which describes the change in CT_max_ relative to the shift in acclimation temperature (Claussen, 1977). Global syntheses indicate that the potential magnitude of acclimatory gains is constrained: most ARR values across ectothermic species fall well below the theoretical maximum of 1, implying only partial compensation for warming (Gunderson & Stillman, 2015; Morley et al., 2019). These limitations appear particularly pronounced in warm‐adapted species, where high baseline heat tolerance may trade off with the potential for further plasticity (Armstrong et al., 2019; Stillman, 2003). Even so, partial adjustment can buffer organisms against short‐term extremes and provide time for longer‐term adaptation (Bush et al., 2016; Morley et al., 2019).

While acclimation capacity reflects the extent of physiological adjustment, survival during rapid warming depends on acclimation dynamics: the rate at which physiological systems respond to changing temperature (Einum et al., 2019; Morley et al., 2019). The speed of response becomes particularly critical during short‐lived extreme events, which often persist for only a few days (Parry‐Wilson et al., 2024). Under such conditions, the outcome depends on a race between rising temperatures and the organism's ability to induce protective acclimation before functional thresholds are exceeded (Morley et al., 2019; Peck et al., 2014). Across ectotherms, a trade‐off has been observed between the rate and magnitude of plasticity, with species with greater acclimation potential often responding more slowly (Burton & Einum, 2025). Because many heatwaves subside before acclimation is complete, physiological protection may remain partial or absent.

Beyond short‐term acclimation, species biogeography also shapes thermal limits. Both upper and lower tolerance limits tend to decline with increasing latitude, likely reflecting adaptation to local temperature regimes (Sunday et al., 2019). In marine ectotherms, these patterns are strongly influenced by sea surface temperature (SST), a key driver of physiological tolerance (Pinsky et al., 2019). Populations in warm, low‐latitude regions generally exhibit higher thermal maxima but live near their physiological limits, making them especially vulnerable to extreme heat events. In contrast, high‐latitude species often maintain broader safety margins yet possess lower absolute heat tolerance, leaving them exposed when rare thermal anomalies exceed these limits (Gunderson & Stillman, 2015; Pinsky et al., 2019). While such trends are well‐established for heat tolerance, similar patterns are not observed for thermal plasticity. Theoretical expectations that organisms from thermally variable, high‐latitude environments should display greater acclimation capacity (Janzen, 1967) are largely unsupported (Gunderson & Stillman, 2015; Stillman, 2003), suggesting limited protection against rapid warming across latitudes (Gunderson & Stillman, 2015; Morley et al., 2019).

In this study, we quantified thermal tolerance and short‐term acclimation capacity in 10 mobile macroinvertebrate species inhabiting a shallow coastal ecosystem in the Northeast Atlantic. These environments experience pronounced temperature variability and are increasingly exposed to more intense, frequent and severe marine heatwaves (Oliver et al., 2018). This is concerning because shallow coastal habitats play a key role in marine ecosystem functioning, supporting services such as nutrient cycling (Attard et al., 2019) and primary production (Duarte et al., 2005). Many of these processes depend on multiple invertebrate species within the same system (Rodil et al., 2022), meaning that the loss of functional groups or keystone species may compromise ecological integrity and habitat resilience (Smith et al., 2023). Given their ecological importance and high risk of thermal exposure, coastal invertebrates provide useful models for examining plasticity and biogeographical drivers of thermal tolerance under rapid ocean warming (Smith et al., 2024).

Specifically, we examined mobile macroinvertebrate taxa from the upper 0–4 m of Northeast Atlantic benthic habitats, including Arthropoda: *Pagurus bernhardus* (common hermit crab), *Praunus flexuosus* (mysid), *Crangon crangon* (brown shrimp), *Carcinus maenas* (European green crab), *Palaemon elegans* (rockpool prawn), *Hemigrapsus takanoi* (Asian shore crab); Mollusca: *Aeolidia papillosa* (grey sea slug), *Tritia nitida* (netted dog whelk), *Littorina littorea* (common periwinkle); and Echinodermata: *Asterias rubens* (common sea star). We exposed wild‐collected individuals to two temperature regimes for 5 days: a control at 15°C, representing ambient surface conditions at the time of collection, and a heatwave treatment 5°C above ambient. After the acclimation, we quantified acute upper thermal limits using CT_max_ tests. Our broad‐scale approach enabled us to test three key predictions: (1) exposure to the simulated heatwave would increase acute upper thermal tolerance across species, indicating short‐term acclimation to warmer conditions (Stillman, 2003; Stillman & Somero, 2000); (2) CT_max_ would increase with the maximum environmental temperatures encountered across species' distributions, reflecting broad‐scale biogeographic constraints on thermal limits; and (3) species occurring at higher latitudes would exhibit lower absolute thermal limits, whereas those occupying warmer or broader ranges would tolerate higher temperatures (Sunday et al., 2019). Because all individuals were collected from a single site, these analyses should be interpreted as species‐level comparisons between experimentally measured CT_max_ and broad‐scale range descriptors, rather than as tests of population‐level latitudinal adaptation. By assessing short‐term acclimation under heatwave conditions, we evaluate both intrinsic thermal limits and plastic capacity in coastal invertebrates, providing insight into their resilience to rapid warming and the broader implications for coastal biodiversity under climate change.

## 2 METHODS

### 2.1 Animal collection and husbandry

We collected individuals on multiple occasions in June 2025 from Bökevik Bay, adjacent to the Kristineberg Marine Research Station, Sweden (58°′24.9″N 11°44′58.5″ E). We captured crustaceans (*C. crangon*, *P. flexuosus*, *C. maenas* and *P. elegans*) using a beach seine in shallow sandy areas. We hand‐netted *A. papillosa*, *H. takanoi*, *L. littorea*, *A. rubens*, *P. bernhardus* and *T. nitida* while snorkelling in the same bay. We also obtained additional *A. rubens* from baited cages suspended from the station's docks.

After capture, we maintained the animals in flow‐through glass aquaria supplied with fjord seawater from 7 m depth, maintaining conditions comparable to the collection site (14.9°C ± 0.3°C; salinity 29.20 ± 0.01 PSU; mean ± SD). We set the photoperiod to 18 h light: 6 h dark to match local conditions. To prevent aggressive interactions and predation, all organisms were housed in species‐specific aquaria, with crab species additionally size‐sorted before housing. We enriched the aquaria with plastic algae and fed the animals daily with brine shrimp (*Artemia salina*). We provide collection dates and holding durations in Table S1.

### 2.2 Acclimation treatments

We conducted acclimation treatments in a temperature‐controlled room at the Kristineberg Marine Research Station, using continuous flow‐through seawater (salinity 29.20 ± 0.01 PSU). We transferred individuals from holding aquaria into food‐grade, semi‐transparent plastic containers (~12.2 L and 5.3 L).

We then exposed the animals to one of two temperature regimes: a control (15.28°C ± 0.26°C), representing ambient conditions or a heatwave treatment (20.14°C ± 0.21°C), ~5°C above ambient. Sea temperature patterns can be found in the supplementary material (Figure S1). We selected this +5°C anomaly to represent a severe but ecologically relevant short‐term warming event for the Skagerrak region, consistent with marine heatwave classification frameworks and recent experimental work in nearby coastal habitats (Hobday et al., 2016; Tamarit Castro et al., 2026). For each species, we assigned three replicate containers per treatment, stocking 10–13 individuals per tank. To prevent thermal shock, we gradually achieved the target heatwave temperature over 24 h by mixing heated and control inflows (Table S1). After ramping, we maintained animals at treatment temperatures for four consecutive days to simulate a short heatwave.

We checked water flow, animal condition and mortality daily. We measured temperature in each container daily using digital thermometers calibrated against a laboratory‐grade reference thermometer. We maintained dim lighting to limit algal growth, using an 18 h light: 6 h dark photoperiod with simulated dawn (50% light, 06:00–08:00) and dusk (50% light, 22:00–23:00).

### 2.3 Upper thermal tolerance trials

After acclimation, we determined CT_max_ using a standardised protocol adapted from Raby et al. (2025). We transferred individuals to a partitioned tank that separated the animal arena from a heating compartment containing a coil heater and circulation pump. We filled the tank with water at the individual's acclimation temperature and continuously aerated it to maintain oxygen saturation. We allowed individuals to habituate for 10 min before initiating heating, then increased the temperature at ~0.3°C min^−1^ (mean ± SD: 0.303 ± 0.007°C min^−1^).

Observers were blinded to treatment for all trials except two nudibranch trials, where blinding was not maintained at the start. To reduce potential bias, we included at least one experienced observer per trial and applied pre‐defined, species‐specific criteria for loss of motor function, selected to reflect functionally equivalent loss of coordinated movement across species (Table 1).

**TABLE 1:** Behavioural responses to acute thermal exposure in 10 macroinvertebrate species from northeast Atlantic shallow coastal habitats.

Species	Phylum	Behavioural response	CT_max_ endpoint
*L. littorea*	Mollusca	Periwinkle snail—Typically remains attached to arena walls; movement increases with warming until the foot fully retracts.	Complete retraction into the shell and detachment from the substrate.
*T. nitida*	Mollusca	Whelk—Highly mobile under control conditions and frequently rights itself; warming leads to unusual stillness while remaining attached.	Failure (or marked difficulty) in righting after flipping; absence of siphon/core retraction to gentle stimulation recorded as secondary confirmation.
*A. papillosa*	Mollusca	Nudibranch—Minimal movement under control conditions and remains mostly stationary during warming.	Inability to self‐right.
*P. bernhardus*	Arthropoda	Hermit crab—Usually active within the shell but does not self‐right using available surfaces. With warming, individuals become agitated and either (1) abandon the shell or (2) fully withdraw and stop responding to touch.	Only complete withdrawal with no tactile response.
*H. takanoi*	Arthropoda	Asian shore crab—Exploratory under control conditions; becomes increasingly agitated with warming and frequently attempts to climb/escape.	Flips onto dorsal side and cannot self‐right.
*C. maenas*	Arthropoda	Green crab—Normally active and exploratory; warming increases agitation (wall climbing, piling), with rapid claw movements near the endpoint.	Flips onto dorsal side and cannot self‐right.
*P. elegans*	Arthropoda	Rockpool shrimp—Active swimmer; warming initially increases swimming/exploration (escape behaviour), followed by dorsoventral curling or flipping.	No response to tactile stimulation (extra care needed for highly active individuals).
*C. crangon*	Arthropoda	Brown shrimp—Actively swims/explores; warming causes brief hyperactivity followed by reduced activity and curling/flipping near the endpoint.	No response to tactile stimulation.
*P. flexuoxux*	Arthropoda	Mysid shrimp—Highly active at ambient temperature; activity declines with warming, with twitching or dorsoventral curling before loss of control.	No response to tactile stimulation.
*A. rubens*	Echinodermata	Sea star—Initially shows limited movement and remains attached; warming induces arm curling and reduced adhesion.	Loss of attachment with arm drop. Individuals fully curled and unresponsive were also scored as LOE even if tube feet remained attached, as attachment can persist after loss of motor function.

*Note*: CT_max_ endpoints were defined as the temperature at which individuals lost motor function.

We conducted two to three trials in parallel, staggering start times by 10–15 min to ensure careful monitoring. We randomised trial order across acclimation treatments and replicates within each species. Once individuals reached loss of motor function, we immediately transferred them to recovery tanks (16°C ± 1°C for control; 20°C ± 1°C for heatwave) and observed them for 30 min. We then assessed recovery, recorded mortality and weighed individuals. After completing the study, we returned all individuals to Bökevik Bay.

Further details on material specifications, brands, sizes and the rationale for methodological choices are provided in the Supporting Information.

### 2.4 Statistical analyses

We performed all statistical analyses and data visualisation using R (version 4.5.1; R Core Team, 2025) through RStudio (version 2025.05.1 + 513; Posit Team, 2025). For the main analyses, we included only individuals that survived the experimental procedures. To assess potential bias, we repeated all analyses including non‐survivors; these sensitivity results are reported in Tables S2 and S3. Survival was high (98.3%), and the few mortalities (*N* = 10) were considered abnormal responses and excluded as outliers. We also excluded the first trial with *P. bernhardus*, as behavioural responses for loss of motor function did not meet pilot study criteria. For all models, we set statistical significance at *p* < 0.05. To account for potential non‐independence among species due to shared ancestry, we repeated the species‐level analyses using phylogenetically informed models as sensitivity tests. Because these analyses were based on only 10 species, and phylogenetic signal was weak and non‐significant for mean CT_max_, ARR and TSM, we interpreted them cautiously and retained the simpler non‐phylogenetic models as the main analyses. Details of the phylogenetic analyses and full model outputs are provided in Tables S4–S6 and Figure S2.

#### 2.4.1 Variation in thermal tolerance

We analysed intra‐ and interspecific variation in thermal tolerance using linear mixed‐effects models (LMMs) fitted with the lmer function (Bates et al., 2015). In the baseline model, we included treatment, species and their interaction as fixed effects, and specified tank identity as a random intercept to account for potential pseudoreplication from shared rearing environments. To assess the effect of body mass, we scaled individual mass and included it as a covariate in additional models. For each species, we fitted models with and without mass and inspected residuals to confirm model adequacy.

We used the lmerTest package (Kuznetsova et al., 2017) for statistical inference, applying Satterthwaite's approximation for degrees of freedom. We checked model assumptions of normality and homoscedasticity using the performance package (Lüdecke et al., 2021). In the full model, we included treatment, species and body mass as fixed effects, with a treatment × species interaction to test for species‐specific acclimation responses. We removed non‐significant interactions involving body mass (*p* > 0.05). We estimated marginal means (EMMs) and pairwise contrasts using the emmeans package (Lenth & Piaskowski, 2017).

#### 2.4.2 Acclimation capacity

For each species, we quantified acclimation as the change in critical thermal maximum, ΔCT_max_, between acclimation treatments and expressed it as ARR following Claussen (1977). We defined ARR as the slope of the relationship between thermal tolerance and acclimation temperature, with:

(1) $$\mathrm{ARR}=\frac{{\text{ΔCT}}_{\text{max}}}{\Delta T}=\frac{{\text{CT}}_{\text{max}}\;\left(20^{{}^{\circ}}C\right)-{\text{CT}}_{\text{max}}\;\left(15^{{}^{\circ}}C\right)}{20-15}$$

where CT_max_ (20°C) and CT_max_ (15°C) are the mean critical thermal maxima for individuals acclimated to the heatwave and control treatments, respectively, and ΔT is the 5°C difference in acclimation temperature.

To determine the precision of ARR estimates, we calculated its sampling variance ($\sigma_{\mathrm{ARR}}^{2}$) using the error‐propagation method, which incorporates the independent variances of the two means (Noble et al., 2022; Pottier et al., 2021, 2024). Because ARR is a ratio involving the difference between two independent CT_max_ values, we summed their variances and scaled by the square of the denominator ($\Delta T^{2}$) (Noble et al., 2022):

(2) $$\sigma_{\mathrm{ARR}}^{2}=\frac{1}{{\left(\Delta T\right)}^{2}}\left({\mathrm{SE}}_{\text{CTmax}\left(20\right)}^{2}+{\mathrm{SE}}_{\text{CTmax}\left(15\right)}^{2}\right)$$

we calculated the standard error of ARR as the square root of this variance:

(3) $${\mathrm{SE}}_{\mathrm{ARR}}=\frac{1}{\Delta T}\sqrt{{\mathrm{SE}}_{\text{CTmax}\left(20\right)}^{2}+{\mathrm{SE}}_{\text{CTmax}\left(15\right)}^{2}}$$

We compared species‐level acclimation capacity using weighted meta‐analytical models implemented in the R package *metafor* (Pottier et al., 2024; Viechtbauer, 2010). We weighted each species' ARR by the inverse of its sampling variance, so estimates with greater precision contributed more to the analysis (Noble et al., 2022).

We also quantified the short‐term change in thermal tolerance for each species, referred to here as the thermal tolerance gain (TT_gain_). TT_gain_ reflects the increase in an organism's upper thermal limit relative to its baseline condition following exposure to an acclimation treatment (De Bonville et al., 2025). It was calculated as follows:

(4) $${\mathrm{TT}}_{\text{gain}}={\mathrm{CT}}_{\max}\;\left(20^{{}^{\circ}}C\right)-{\mathrm{CT}}_{\max}\;\left(15^{{}^{\circ}}C\right)$$

where CT_max_ (20°C) and CT_max_ (15°C) are the mean critical thermal maxima for individuals acclimated to the heatwave and control treatments, respectively.

#### 2.4.3 Species distribution data

We obtained species distribution data from the Global Biodiversity Information Facility (GBIF.org, 2025; Table S8; Figure S3). We restricted distributions to the European range for all species except the invasive crab *H. takanoi*, for which we used the global range to better represent its thermal niche. We calculated geographic range descriptors, minimum latitude, maximum latitude and latitudinal breadth using geospatial tools in R (packages sf; Pebesma, 2016; and rnaturalearth; Massicotte & South, 2017). These predictors describe broad species‐level distributional patterns rather than the local collection environment of the experimental individuals.

We examined relationships between thermal tolerance, acclimation capacity and geographic position using two approaches. First, we analysed the relationship between latitude and CT_max_ using linear mixed‐effects models. In these models, individual CT_max_ values (grouped by acclimation treatment) served as the response variable. We included one geographic metric as a fixed effect and specified species identity and tank ID as random intercepts to account for species‐level variation and potential tank‐level dependencies. We conducted inference using Satterthwaite's approximation for degrees of freedom (lmerTest; Kuznetsova et al., 2017) and verified model assumptions using residual diagnostics (performance; Lüdecke et al., 2021). Second, we assessed geographic variation in ARR using meta‐regression models implemented in the *metafor* package (Viechtbauer, 2010). These models used species‐level ARR estimates and their sampling variances as effect sizes, with latitude included as a moderator.

#### 2.4.4 Sea surface temperature data

We assessed correlations between SST and thermal tolerance using Bio‐ORACLE environmental data layers (Assis et al., 2024; Tyberghein et al., 2012). For each species' occurrence we extracted six SST descriptors to capture different dimensions of local thermal regimes: long‐term maximum and minimum, instantaneous maximum and minimum, mean, and range (average annual difference between maximum and minimum SST) (Figure S5). These SST descriptors were used to characterise the thermal environments encompassed by each species' distribution, not the recent developmental or acclimation history of the individuals tested.

We fitted linear mixed‐effects models with CT_max_ as the response variable, treatment and one SST descriptor as fixed effects, and species identity and tank ID as random intercepts. This structure accounted for interspecific variation and non‐independence among individuals from the same tank. We conducted inference using Satterthwaite's approximation (lmerTest; Kuznetsova et al., 2017) and evaluated model assumptions using residual diagnostics (performance; Lüdecke et al., 2021).

We analysed ARR separately using meta‐regression models (metafor; Viechtbauer, 2010), treating species‐level ARR estimates and their sampling variances as effect sizes and including SST descriptors as moderators.

In addition to model analyses, we used the same Bio‐ORACLE dataset to estimate thermal safety margins (TSM) for each individual under the different acclimation treatments. TSM quantifies the distance between an organism's upper thermal tolerance limit and the highest environmental temperature it typically experiences. We calculated thermal safety margin as follows:

(5) $$\mathrm{TSM}={\mathrm{CT}}_{\max}-T_{\max,\mathrm{env}}$$

where CT_max_ is the individual's critical thermal maximum (°C) determined experimentally, and T_max,env_ is the local maximum sea surface temperature (°C) derived from Bio‐ORACLE's instantaneous maximum SST layer.

### 2.5 Ethical approval

Under Swedish legislation implementing EU Directive 2010/63/EU, as well as the Swedish Animal Welfare Act (SFS 2018:1192), the species used in this study are not subject to mandatory ethical review.

## 3 RESULTS

### 3.1 Upper thermal tolerance across species

We observed that the critical thermal maximum (CT_max_, means ± SE) increased in all species following simulated heatwave exposure (20°C) compared with control conditions (15°C) with significant differences detected between the two treatments (*p* < 0.001; Figure 1 and Table 2). However, species differed in their thermal limits. Under control conditions, *A. papillosa* exhibited a CT_max_ of 27.57°C ± 0.11°C, while *H. takanoi* reached 36.18°C ± 0.13°C. After heatwave acclimation, we found a similar breadth, with CT_max_ spanning from 28.24°C ± 0.22°C (*A. papillosa*) to 37.27°C ± 0.16°C (*H. takanoi*) (Table S9; Figure 1).

**FIGURE 1:**
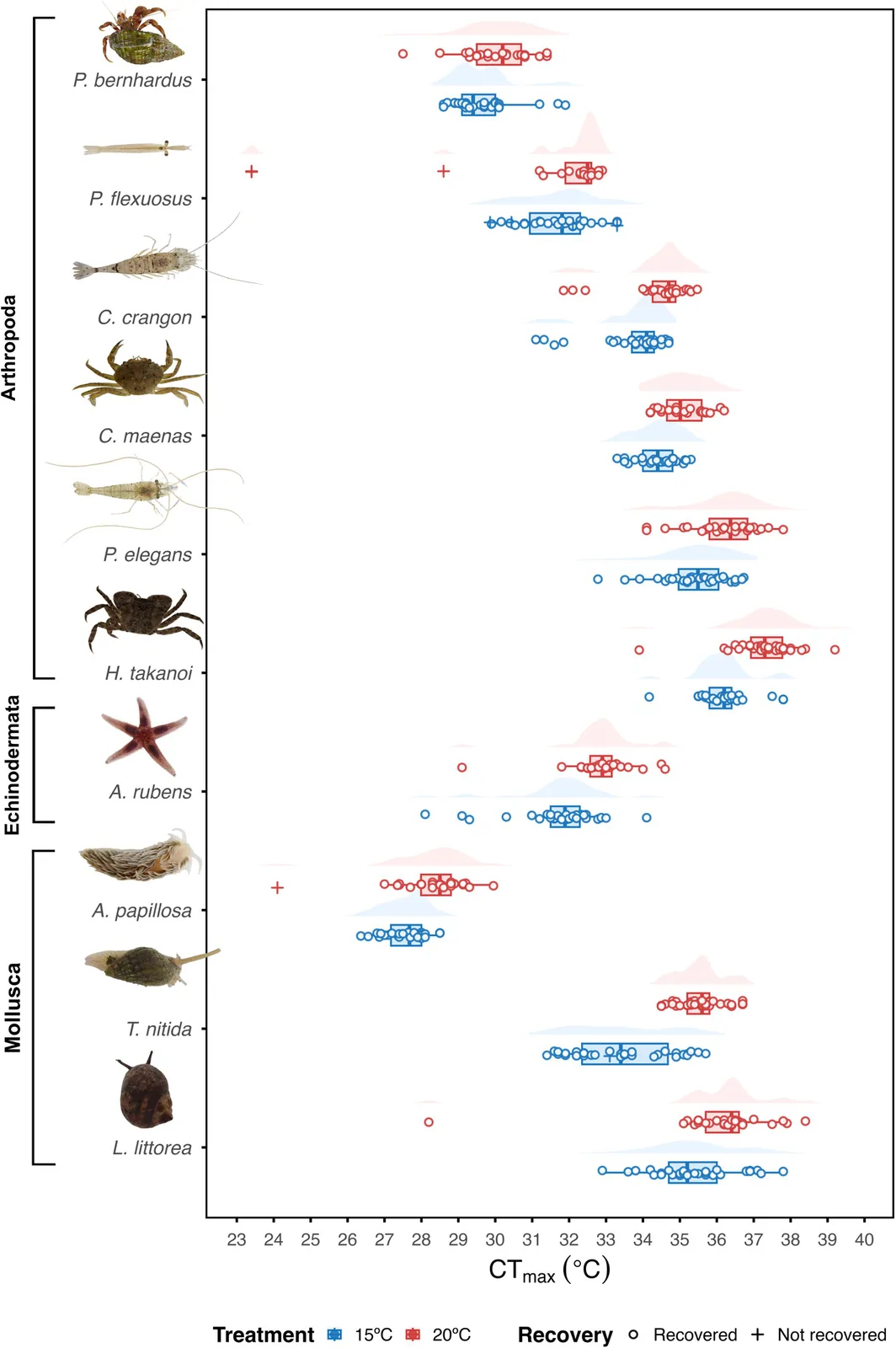
CT_max_ of 10 marine invertebrate species under two short acclimation treatments. Individuals were acclimated to either 15°C (blue; ambient control) or 20°C (red; simulated heatwave). Each point represents the CT_max_ of an individual. Kernel density curves show the full distributions for each treatment, with boxplots overlaid to illustrate the interquartile range (IQR) for recovered individuals. Recovery status 30 min after the trial is indicated by point shape (circles = recovered; cross = not recovered).

**TABLE 2:** Analysis of variance table for the two linear mixed‐effects models of CT_max_.

Response	Predictors	df	Den df	F‐value	*p*‐value	*R* ^2^m	*R* ^2^c
CTmax (model 1) (*N* = 563)	**Treatment**	1	60.003	6.255	**0.01513**	0.893	0.904
	**Treatment:species**	18	55.888	135.71	**<2e−16**	0.893	0.904
CTmax (model 2) (*N* = 563)	**Treatment**	1	69.23	5.503	**0.02186**	0.859	0.905
	**Treatment:species**	18	82.60	38.770	**<2e−16**	0.859	0.905
	Species:mass	10	343.28	1.277	0.2415	0.859	0.905

*Note*: The first model tested the effect of treatment and its interaction with species, ignoring overall species differences and focusing on whether treatment effects differed between species. The second model tested the effects of treatment, species and body mass and their interactions. *R* ^2^m represents the marginal *R* ^2^, while *R* ^2^c represents the conditional *R* ^2^. Significant predictors (*p* < 0.05) are shown in bold.

The magnitude of thermal plasticity differed significantly across species (*p* < 0.001), and we observed particularly strong responses in *T. nitida* (*p* < 0.0001; ARR = 0.42; 95% CI = 0.04, 0.23), *A. rubens* (*p* = 0.0049; ARR = 0.23; 95% CI = 0.12, 0.35), *H. takanoi* (*p* = 0.0142; ARR = 0.22; 95% CI = 0.13, 0.30) and *L. littorea* (*p* = 0.0282; ARR = 0.14; 95% CI = 0.14, 0.29). Notably, these were the only species where the change in CT_max_ following heatwave exposure was statistically significant.

We detected the highest acclimation capacity (TT_gain_) in *T. nitida*, which increased CT_max_ by 2.18°C (ARR = 0.42; 95% CI = 0.04, 0.23). In contrast, *P. bernhardus* displayed minimal plasticity (TT_gain_ = 0.42°C; ARR = 0.08; 95% CI = −0.01, 0.18). Crustaceans exhibited divergent strategies: while *C. maenas* and *P. elegans* maintained high baseline CT_max_ (>34°C), they exhibited low plasticity (ARR = 0.14–0.16; 95% CI = 0.07, 0.20–0.06, 0.25). Conversely, *H. takanoi* combined high absolute tolerance with moderate acclimation capacity (ARR = 0.22; 95% CI = 0.13, 0.30) (Figure 2; Table S9). Among molluscs, we identified strong plasticity in *T. nitida* (TT_gain_ = 2.18; ARR = 0.42; 95% CI = 0.32, 0.53), while *L. littorea* showed high baseline tolerance (35.40°C ± 0.21°C) but limited acclimation capacity (TT_gain_ = 0.69°C; ARR = 0.14; 95% CI = −0.01, 0.29). *A. papillosa* consistently exhibited the lowest thermal limits across treatments, despite a modest increase in acclimation (ARR = 0.15; 95% CI = 0.04, 0.23). The echinoderm *A. rubens* displayed intermediate plasticity (TT_gain_ = 1.17°C; ARR = 0.23; 95% CI = 0.12, 0.35) (Figure 2; Table S9). The random effects meta‐analysis confirmed this overall plasticity (*p* < 0.001; mean ARR = 0.18; 95% CI = 0.12, 0.24), demonstrating that, on average, a + 5°C increase in acclimation temperature raised CT_max_ by 0.9°C. Finally, we found no statistically significant association between wet mass and CT_max_ (*p* = 0.2415) (Figure S4; Table 2).

**FIGURE 2:**
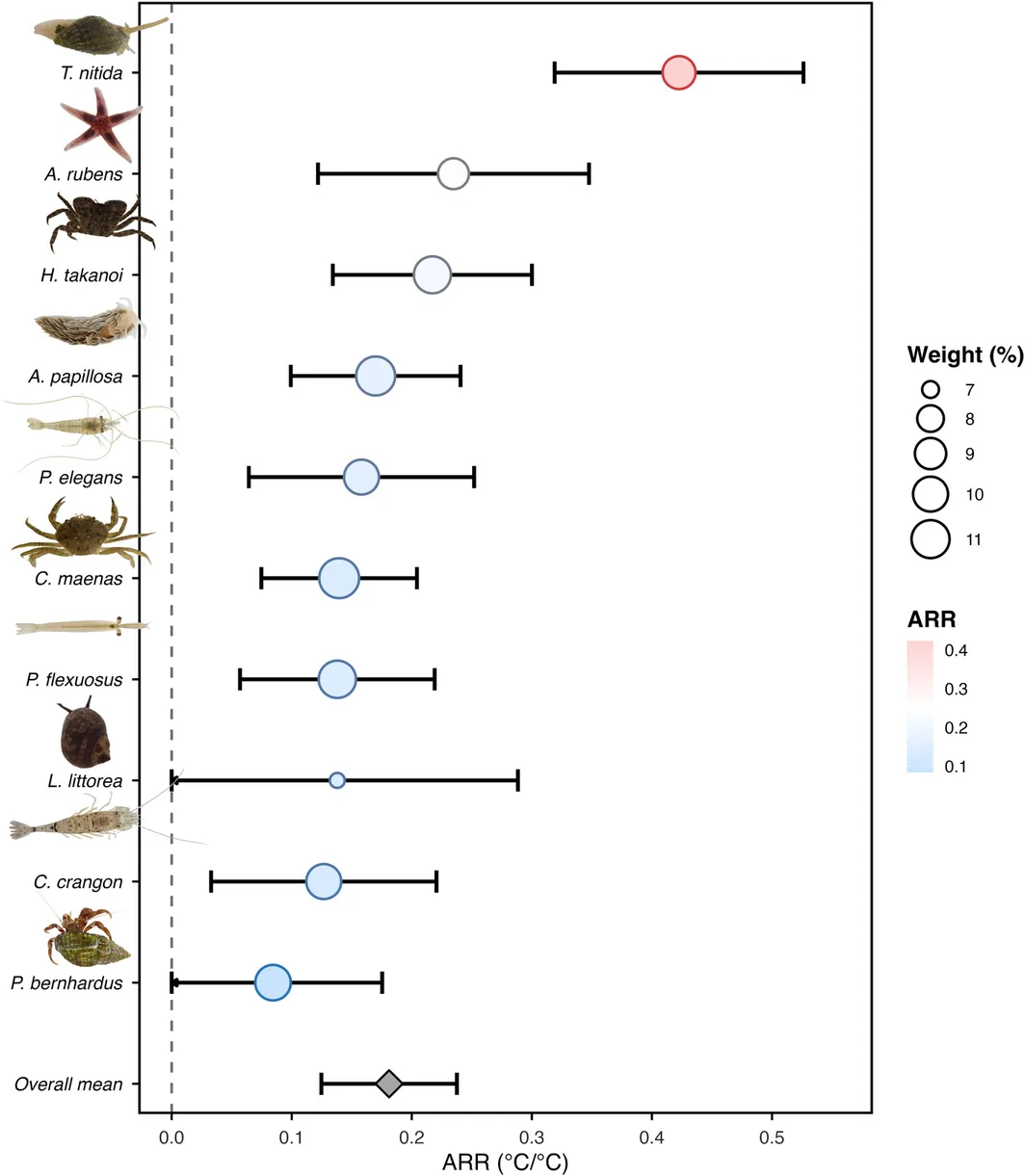
Species‐specific estimates of ARR (°C/°C). Points represent the estimated ARR for each species, with point size scaled to their meta‐analytic weight and fill colour indicating ARR magnitude. Horizontal bars show 95% confidence intervals, and the diamond represents the overall pooled mean estimate.

### 3.2 Geographic distribution and thermal tolerance

We found evidence for correlations between geographic distribution and heat tolerance, but not for acclimation capacity (Figure 3; Table 3). In our analysis, CT_max_ trended downwards with both increasing latitudinal range and maximum latitude, although these trends were not statistically significant. Specifically, for each degree increase in maximum latitude, CT_max_ declined by 0.15°C (95% CI = −0.404, 0.094; *p* = 0.262), and for each additional degree of latitudinal range, CT_max_ dropped by 0.14°C (95% CI = −0.081, 0.366; *p* = 0.247). While we detected a statistically significant negative association between CT_max_ and minimum latitude (95% CI = −0.431, 0.114; *p* = 0.0097), there were no relationships between ARR and latitudinal range (95% CI = −0.012, 0.005; *p* = 0.404), minimum latitude (95% CI = −0.010, 0.007; *p* = 0.7486) or maximum latitude (95% CI = −0.015, 0.003; *p* = 0.1967). Across all models, the treatment effect on CT_max_ remained highly significant (*p* < 0.001), confirming that simulated heatwave exposure consistently increased thermal limits irrespective of geographic distribution.

**FIGURE 3:**
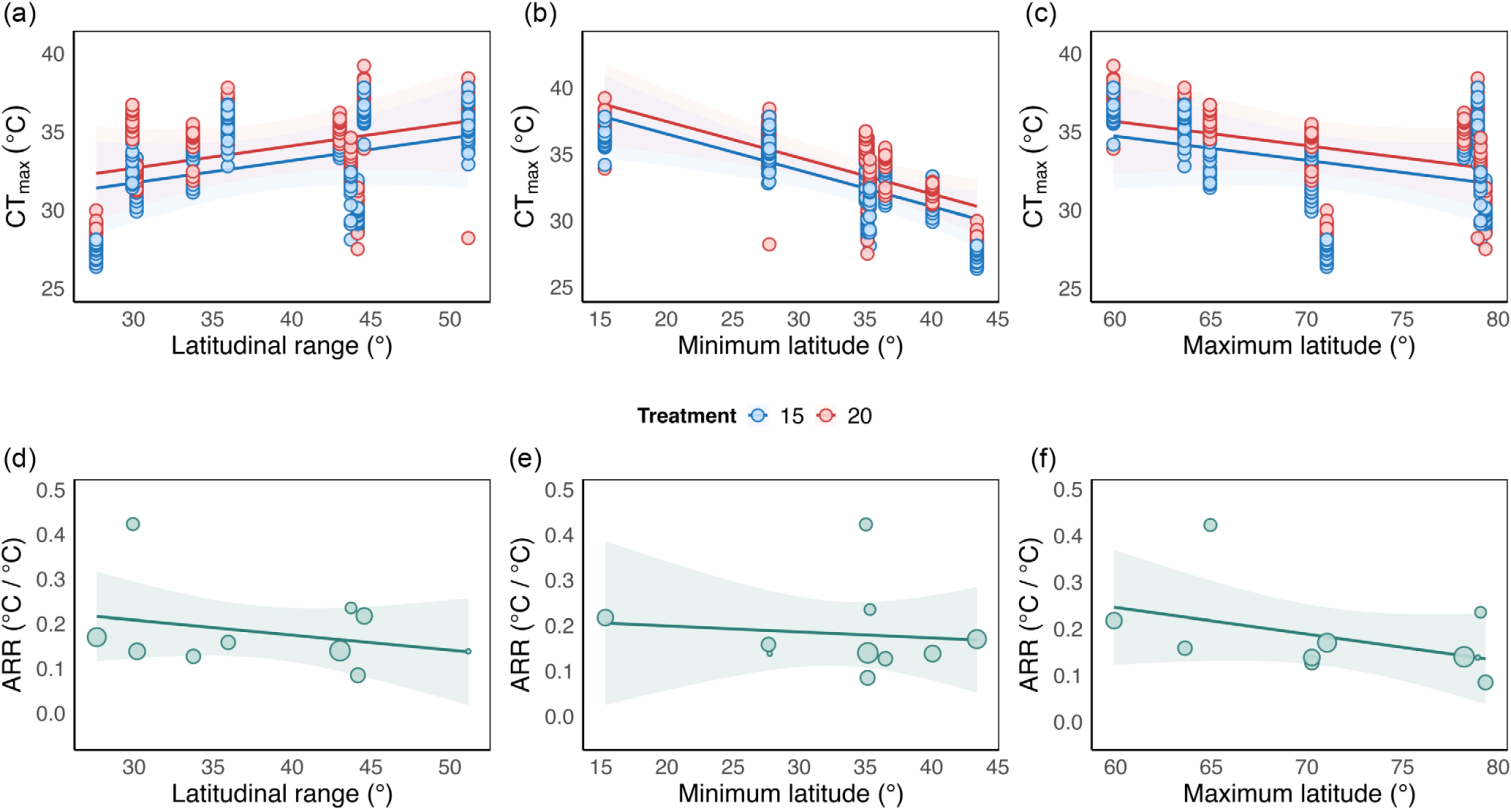
Predicted CT_max_ and ARR for three geographic predictors across the latitudinal ranges of 10 marine invertebrate species. Panels (a–c) show predicted CT_max_ from linear mixed‐effects models including treatment (blue = 15°C control, red = 20°C) and one geographic predictor as fixed effects. Lines represent model predictions, shaded ribbons indicate 95% confidence intervals, and points show individual raw data. Panels (d–f) show predicted ARR from random effects meta‐regressions fitted separately. Lines are model predictions, shaded ribbons show 95% confidence intervals, and points represent study‐level effect sizes, with circle size proportional to precision (1/SE).

**TABLE 3:** Results of linear mixed‐effects models and meta‐analytic mixed‐effects models testing the effects of geographic distribution metrics on CT_max_ and ARR.

Response	Predictors	Estimate	95% CI	df	*p*‐value	*R* ^2^m	*R* ^2^c	*R* ^2^
CTmax	**Intercept**	32.92	31.232–34.610	8.04	**<0.001**	0.903	0.142	
	**Treatment (20°C)**	0.94	0.780–1.103	553.01	**<0.001**			
	Latitudinal range	0.14	−0.081 to 0.366	8.00	0.247			
	**Intercept**	32.99	31.808–34.182	8.07	**<0.001**	0.895	0.494	
	**Treatment (20°C)**	0.94	0.780–1.102	553.02	**<0.001**			
	**Minimum latitude**	−0.27	−0.431 to −0.114	7.99	**0.0097**			
	**Intercept**	32.95	31.256–34.649	8.03	**<0.001**	0.903	0.141	
	**Treatment (20°C)**	0.94	0.780–1.103	552.01	**<0.001**			
	Maximum latitude	−0.15	−0.404 to 0.094	7.99	0.262			
ARR	**Intercept**	0.17	0.111–0.248	6.068	**<0.001**			0
	Latitudinal range	−0.003	−0.012 to 0.005	−0.831	0.404			
	**Intercept**	0.1817	0.111–0.252	5.9509	**<0.001**			0
	Minimum latitude	−0.0013	−0.010 to 0.007	−0.3317	0.7486			
	**Intercept**	0.1794	0.116–0.242	6.5932	**<0.001**			14.60
	Maximum latitude	−0.0057	−0.015 to 0.003	−1.4083	0.1967			

*Note*: For CT_max_, models included treatment and one geographic predictor as fixed effects. For ARR, separate meta‐analytic models were fitted using species as the grouping factor and each geographic metric as a moderator. For CT_max_ mixed‐effects models, *R* ^2^m (marginal *R* ^2^) indicates variance explained by fixed effects, whereas *R* ^2^c (conditional *R* ^2^) indicates variance explained by fixed and random effects combined. For ARR models, *R* ^2^ indicates the proportion of total heterogeneity explained by moderator variables. Significance predictors (*p* < 0.05) are shown in bold.

#### 3.2.1 Sea surface temperature and thermal tolerance

We found that sea surface temperature showed weak, non‐significant associations with absolute thermal limits for the species we studied (Figure S6; Table S9). Our analysis revealed that CT_max_ did not significantly relate to mean SST (95% CI = −0.625, −3.430; *p* = 0.212), minimum SST (95% CI = −1.240, −1.69; *p* = 0.77), long‐term minima (95% CI = −1.232, −1.884; *p* = 0.63) or SST range (95% CI = −0.596, −1.407; *p* = 0.45). We observed positive trends with maximum SST (95% CI = −0.259, −2.293; *p* = 0.15) and long‐term maxima (95% CI = −0.197, −2.620; *p* = 0.13), but these did not show statistical significance.

ARR showed no significant relationships with long‐term maxima (95% CI = −0.054 to 0.054, *p* = 0.99), long‐term minima (95% CI = −0.033 to 0.064, *p* = 0.53), maximum SST (95% CI = −0.049 to 0.047, *p* = 0.80), mean SST (95% CI = −0.051 to 0.091, *p* = 0.58), minimum SST (95% CI = −0.033 to 0.059, *p* = 0.59) or SST range (95% CI = −0.040 to 0.026, *p* = 0.68).

Thermal safety margins differed among species (Figure 4; Table S10). Under both acclimation temperatures, species such as *H. takanoi* and *A. papillosa* showed some of the narrowest safety margins. In contrast, *L. littorea* and *C. maenas* consistently displayed among the widest thermal safety margins. This overall pattern of species‐level differences was similar in both treatments, with only modest shifts in the relative ranking of species.

**FIGURE 4:**
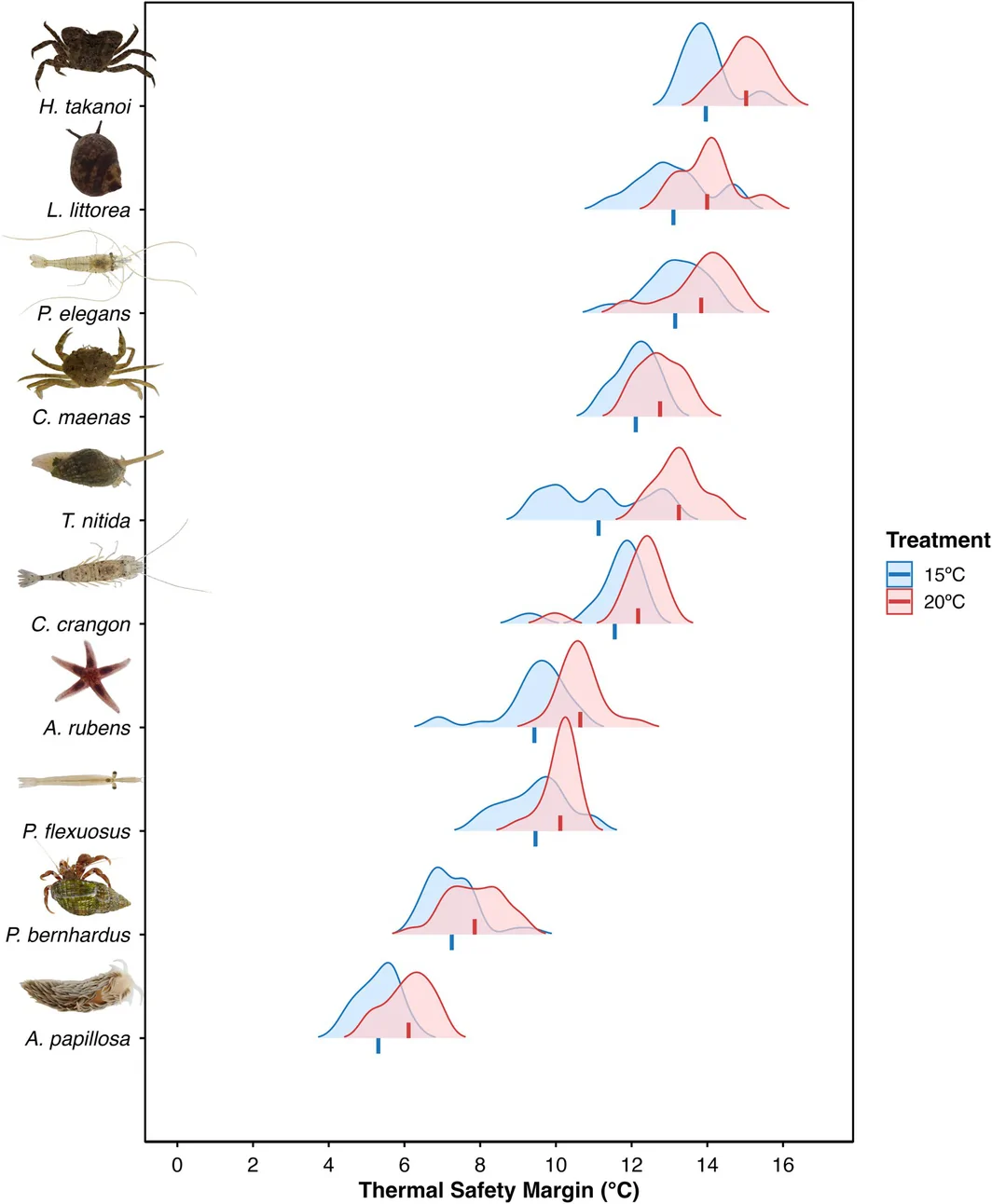
TSM for 10 marine invertebrate species under two acclimation treatments. For each species, kernel density ridges show the distribution of individual TSM values at 15°C (blue) and 20°C (red). Curves represent smoothed distributions of TSM within each treatment, and shaded areas indicate density.

## 4 DISCUSSION

Understanding how marine invertebrates respond to heat stress is essential for predicting their vulnerability to ocean warming. In this study, we investigated the thermal responses of mobile macroinvertebrates inhabiting Northeast Atlantic shallow coastal habitats, ecosystems increasingly exposed to rising temperatures. Ten common species were maintained under both control and short‐term heatwave conditions to assess their ability to adjust upper thermal limits.

Exposure to a simulated heatwave increased the upper thermal limits in all species (Figure 1). These responses align with the broader understanding that thermal acclimation enables organisms to adjust performance following sustained or brief exposure to novel thermal conditions (Angilletta, 2009; Jutfelt et al., 2024). Similar patterns have been reported across ectotherms, with warmer acclimation histories associated with higher CT_max_ values (e.g. Cereja, 2020; Madeira et al., 2019). However, despite this overall increase, the magnitude of change was modest. Across species, CT_max_ increased by an average of only 0.9°C, indicating a rapid yet limited physiological adjustment that may be insufficient to counter acute thermal stress.

In our study, nine species showed an increase in CT_max_ of more than 0.6°C after 5 days of heatwave exposure, but only three exceeded a 1°C increase, and just one, the gastropod *T. nitida*, exceeded 2°C. Although these gains demonstrate rapid physiological adjustment, their magnitude remained small relative to the imposed warming. This pattern aligns with evidence that acclimatory shifts in marine ectotherms are constrained (Armstrong et al., 2019; Gunderson & Stillman, 2015). For example, marine crustaceans often show only slight increases in thermal limits even after extended warming (Vinagre et al., 2016). Nevertheless, the positive CT_max_ shifts observed here reinforce that thermal acclimation provides a short‐term buffer against acute warming.

Despite these increases, their magnitude was modest relative to the imposed warming. A 5°C rise in acclimation temperature for ~4 days produced an average TT_gain_ of only 0.9°C (ARR = 0.18) (Figure 2). Our values are lower than those found in previous comparative syntheses: Gunderson and Stillman (2015) reported an average ARR of 0.32 for ectothermic animals, while Pottier et al. (2022) found a mean ARR of 0.20 in developing aquatic invertebrates. However, these meta‐analytic averages integrate data from studies using a wide range of acclimation durations and temperature differentials, both of which can influence estimates of thermal plasticity. Within this context, the relatively low ARR values observed here may reflect either lower‐than‐expected acclimation potential in our species or the limited duration of the acclimation period. Such short exposure likely constrained plastic responses, as many ectotherms require days to weeks to reach maximal acclimation (Burton & Einum, 2025; De Bonville et al., 2025). However, our results may also reflect the absolute thermal regime used. Our heatwave temperature may not be sufficiently stressful to elicit maximum plasticity in some species with high baseline thermal tolerance. For cold adapted species, the 20°C acclimation temperature may already approach their upper physiological limits, imposing a thermal ceiling that constrains further gains in heat tolerance (Jutfelt et al., 2024; Morley et al., 2019). However, if a ceiling effect was the dominant constraint, a clear inverse relationship between baseline thermal tolerance and plasticity would be expected. Yet species with higher CT_max_ did not consistently display the lowest ARR, nor did they exhibit plasticity that correlated strongly with biogeography, suggesting that thermal ceilings alone cannot explain the overall low ARR (Stillman, 2003; Vinagre et al., 2019). Instead, the short exposure duration likely remains an important constraint, and because this design reflects ecologically realistic heatwave development, the limited thermal adjustment observed here points to a potential vulnerability: if species cannot increase acute upper heat tolerance within a few days, they may be unable to withstand future heatwaves.

Although a trade‐off between inherent tolerance and acclimation capacity has often been proposed (Gunderson & Stillman, 2015; Vinagre et al., 2019), with higher CT_max_ values associated with lower plasticity, our findings provide little support for this pattern. Crabs maintained high baseline CT_max_ yet showed only moderate plasticity, whereas the whelk combined moderate baseline tolerance with the highest acclimation response. Conversely, low‐tolerance species such as the nudibranch exhibited minimal plasticity. This suggests that the relationship between tolerance and plasticity is more complex than a simple trade‐off and may vary with ecology, evolutionary history and proximity to physiological limits (Ern et al., 2023). In addition, previously reported trade‐offs may be exaggerated by statistical artefacts further highlighting that this relationship is not universal (Gunderson, 2023; Van Heerwaarden & Kellermann, 2020).

Geographic variation in temperature has been recognised as a major driver of thermal limits in ectotherms (Janzen, 1967; Sunday et al., 2019). According to the Climate Variability Hypothesis (Janzen, 1967), species from temperate regions, which experience greater seasonal fluctuations, should evolve broader tolerance ranges, whereas tropical species, exposed to more stable but consistently warmer conditions, are expected to develop higher yet narrower thermal tolerance ranges (Jensen et al., 2017; Stillman & Somero, 2000). We found a strong, statistically significant negative relationship between CT_max_ and minimum latitude. In contrast, while maximum latitude and total range size showed correlations with CT_max_, these were highly variable and not statistically significant (Figure 3). Our results are consistent with the ‘warm‐edge’ hypothesis: the low‐latitude edge of a species' range is often where populations face the most significant thermal stress, exposing them to maximum environmental temperatures and placing the strongest selection on heat tolerance (MacArthur, 1984; Sunday et al., 2019). Conversely, the cold edge of a species' distribution is more strongly shaped by cold tolerance, which helps explain why CT_max_ correlates more tightly with minimum than maximum latitude. However, because all individuals were collected from a single site, these patterns reflect species‐level biogeographic correlations rather than evidence of local thermal adaptation.

Interestingly, while latitude showed a clear signal, we did not observe significant correlations between CT_max_ and broad‐scale SST metrics. This suggests that biogeographical factors associated with latitude beyond mean temperature, such as microclimatic variability, play a stronger role in shaping thermal limits. Moreover, latitude integrates multiple environmental dimensions, seasonality, photoperiod and the frequency and intensity of heat extremes, which together influence the evolution of thermal tolerance (Sunday et al., 2019). It also acts as a proxy for other ecological gradients, as species across latitudes encounter distinct combinations of stressors such as salinity fluctuations, oxygen limitation, desiccation and predation pressure. Adaptation conditions can generate physiological cross‐tolerance, where selection for one stressor incidentally enhances resistance to others, a phenomenon described as cross‐protection (Rodgers & Gomez Isaza, 2023). The absence of a strong CT_max_–SST relationship, therefore, highlights that species' thermal limits are shaped not by temperature alone, but by the complex, overlapping selection regimes that covary with geography. In addition, CT_max_ measures acute upper thermal tolerance during a gradual temperature ramp, so it may not capture all aspects of how species respond to thermal stress over time. Finally, because all individuals were collected from a single site, broad‐scale SST metrics may not capture the fine‐scale thermal conditions experienced in shallow habitats.

The invasive crab had a notable influence on our geographic analyses, particularly on the relationship between CT_max_ and minimum latitude. Because *H. takanoi* is distributed broadly, including into some of the warmest, low‐latitude zones of our dataset, and exhibits the highest CT_max_ out of the species tested, it anchored the regression at the low‐latitude, high‐tolerance end (Figure 3). This observation reflects a broader phenomenon in invasion ecology. Invasive species are often characterised by broad environmental tolerances and high phenotypic plasticity, traits that facilitate persistence under extreme or variable conditions and support establishment in novel habitats under climate change (Seebacher et al., 2015). In our analyses, *H. takanoi* exemplifies this pattern, elevating observed upper thermal tolerance limits and strengthening apparent latitude‐related trends. This result highlights that the inclusion of invasive taxa can actively reshape biogeographic interpretations and further suggests that ongoing warming may promote the continued expansion and dominance of eurythermal invaders (Sardain et al., 2019).

ARR showed no clear relationship with geographic variables or SST. This aligns with previous work, which suggests that plasticity is more influenced by local environmental conditions, microhabitat use and phylogenetic evolutionary constraints than by broad geographic trends or climatic averages (De Jong, 2005). This is particularly relevant for our study species, which inhabit coastal and estuarine environments characterised by high environmental heterogeneity (Bitter et al., 2021). These habitats experience sharp heterogeneity in temperature and wave exposure, often occurring over the scale of a few meters. Such fine‐scale environmental heterogeneity creates intense selective pressures that can drive adaptive divergence among populations, sometimes even within the same site (Hudson et al., 2019). Global syntheses further indicate that strong latitudinal trends in acclimation capacity emerge primarily when species from thermally extreme regions are included. Morley et al. (2019) reported particularly high ARRs in polar arthropods and aquatic invertebrates, while most temperate and subpolar species exhibit limited plasticity, suggesting that pronounced geographic patterns arise only across very broad climatic scales. Because our dataset spans temperate to subpolar zones excluding true polar taxa, the absence of a latitudinal signal in ARR likely reflects this limited climatic range rather than a true absence of spatial structuring in plasticity.

Our results show that physiological plasticity is relatively limited among most species, with ARR values rarely exceeding 0.3. However, high plasticity is not equally necessary across taxa. Most species maintain a substantial thermal safety margin between their CT_max_ and maximum habitat temperatures, suggesting some present‐day resilience to warming (Vinagre et al., 2019). However, rapid‐ramping CT_max_ estimates likely overstate the temperatures animals can endure for prolonged periods during natural heat events, implying that realised ecological safety margins may be considerably smaller. Concern arises, however, for species with both low plasticity and narrow safety margins. In our dataset, the nudibranch and the hermit crab exhibited some of the lowest ARRs and the smallest thermal safety margins. This combination of limited plastic capacity and minimal thermal buffer leaves these species with little physiological scope to adjust or endure further warming (Armstrong et al., 2019). A growing body of evidence indicates that species with both low thermal safety margins and low thermal plasticity are especially vulnerable to acute mortality and local extirpation during extreme thermal events, with population declines already documented (Parry‐Wilson et al., 2024; Vinagre et al., 2016).

Overall, our findings reinforce the well‐established view that phenotypic plasticity alone provides only partial protection against climate‐driven warming in ectotherms and seldom compensates fully for extreme heat events (Armstrong et al., 2019; Gunderson & Stillman, 2015; Morley et al., 2019). Species persistence will depend on a combination of traits and conditions, sufficient thermal safety margins, access to microhabitat refugia, rapid behavioural adjustments and over longer timescales, evolutionary adaptation (Donelson et al., 2019; Seebacher et al., 2015). As climate change accelerates and heatwaves intensify, species with narrow safety margins and limited plasticity are likely to be most at risk of acute mortality and local extinction. This vulnerability may be amplified in dynamic coastal systems, where access to thermal refugia is constrained, and microclimatic extremes are becoming more frequent and severe.

## AUTHOR CONTRIBUTIONS

Fredrik Jutfelt, Elena Tamarit‐Castro, Leon Green, Mafalda Tomás and Timothy D. Clark conceived the ideas. Mafalda Tomás, Elena Tamarit‐Castro, Elizabeth C. Hoots, Luis L. Kuchenmüller, Tamzin A. Blewett, Ivan Cadonic, Patrice Pottier, Alexandro Autiero, Leon Green, Zara‐Louise Cowan, Juliane Vigneault, Vincent Mélançon, Dustin Doty, Sidney Martin and Maryane Gradito conducted the investigation and collected the data. Mafalda Tomás and Patrice Pottier performed the formal analyses. Mafalda Tomás, Patrice Pottier and Leon Green curated and visualised the data and drafted the manuscript. Leon Green and Fredrik Jutfelt supervised the study. Fredrik Jutfelt and Tamzin A. Blewett acquired the funding. All authors reviewed and edited the manuscript and approved the final version for publication.

## CONFLICT OF INTEREST STATEMENT

The authors declare no conflict of interest.

## STATEMENT ON INCLUSION

This study was conducted as part of an international collaboration. Researchers based in the country where the study was conducted were involved in both the planning of the study and the fieldwork, bringing important local knowledge to the project. All authors contributed from the early stages of the research, helping shape the study design and approach.

## Supporting information


**Table S1.** Experimental design and acclimation conditions for intertidal species exposed to 15°C and 20°C temperature treatments.
**Table S2.** Sensitivity analysis of thermal tolerance (CT_max_) based on linear mixed‐effects models that include all individuals, including those that did not recover 30 min post‐trial.
**Table S3.** Model comparison results for the main and sensitivity linear mixed‐effects models of thermal tolerance (CT_max_), generated using the performance package.
**Table S4.** Phylogenetic signal in main responses.
**Table S5.** Main thermal trait models with and without phylogenetic correction.
**Table S6.** Species‐level predictor models with and without phylogenetic correction.
**Table S7.** Summary of thermal tolerance metrics CT_max_ and acclimation response ratio (ARR) for each species included in the experiment.
**Table S8.** Summary of the geographical distribution of selected marine species based on occurrence records from the GBIF database.
**Table S9.** Results of linear mixed‐effects models and meta‐analytic mixed‐effects models testing the effects of SST metrics on CT_max_ and ARR, respectively.
**Table S10.** Thermal safety margins (TSM) across species and acclimation treatments.
**Figure S1.** Seasonal and short‐term thermal variability at 1 m depth in Gullmarsfjorden from 2020 to 2025.
**Figure S2.** Phylogenetic relationships among the study species.
**Figure S3.** Mean sea surface temperature (SST;°C) across European waters from 2010 to 2020, overlaid with occurrence records for 10 marine invertebrate species.
**Figure S4.** Effect of body mass on CT_max_ across two treatment groups (control and heatwave +5°C) for all species included in the experiment.
**Figure S5.** Spatial summary of sea surface temperature (SST, °C) across European waters for the period 2010–2020.
**Figure S6.** Relationships between CT_max_ (panels A–F) and ARR (panels G–L) and six sea surface temperature (SST) metrics across species.

## Data Availability

All data supporting the results of this study are available in the Zenodo repository: https://doi.org/10.5281/zenodo.19691547 (Tomás et al., 2025).

## References

[jane70332-bib-0001] Angilletta, M. J. (2009). Thermal adaptation. Oxford University Press. 10.1093/acprof:oso/9780198570875.001.1

[jane70332-bib-0002] Armstrong, E. J. , Tanner, R. L. , & Stillman, J. H. (2019). High heat tolerance is negatively correlated with heat tolerance plasticity in nudibranch mollusks. Physiological and Biochemical Zoology, 92(4), 430–444. 10.1086/704519 31192766

[jane70332-bib-0003] Assis, J. , Fernández Bejarano, S. J. , Salazar, V. W. , Schepers, L. , Gouvêa, L. , Fragkopoulou, E. , Leclercq, F. , Vanhoorne, B. , Tyberghein, L. , Serrão, E. A. , Verbruggen, H. , & De Clerck, O. (2024). bio‐oracle v3.0. Pushing marine data layers to the cmip6 earth system models of climate change research. Global Ecology and Biogeography, 33(4), e13813. 10.1111/geb.13813

[jane70332-bib-0004] Attard, K. M. , Rodil, I. F. , Glud, R. N. , Berg, P. , Norkko, J. , & Norkko, A. (2019). Seasonal ecosystem metabolism across shallow benthic habitats measured by aquatic eddy covariance. Limnology and Oceanography Letters, 4(3), 79–86. 10.1002/lol2.10107

[jane70332-bib-0005] Bates, D. , Mächler, M. , Bolker, B. , & Walker, S. (2015). Fitting linear mixed‐effects models using lme4. Journal of Statistical Software, 67(1), 1–48. 10.18637/jss.v067.i01

[jane70332-bib-0006] Bitter, M. C. , Wong, J. M. , Dam, H. G. , Donelan, S. C. , Kenkel, C. D. , Komoroske, L. M. , Nickols, K. J. , Rivest, E. B. , Salinas, S. , Burgess, S. C. , & Lotterhos, K. E. (2021). Fluctuating selection and global change: A synthesis and review on disentangling the roles of climate amplitude, predictability and novelty. Proceedings of the Royal Society B: Biological Sciences, 288(1957), 20210727. 10.1098/rspb.2021.0727 PMC8385344 34428970

[jane70332-bib-0007] Burton, T. , & Einum, S. (2025). High capacity for physiological plasticity occurs at a slow rate in ectotherms. Ecology Letters, 28(1), e70046. 10.1111/ele.70046 PMC11744337 39829299

[jane70332-bib-0008] Bush, A. , Mokany, K. , Catullo, R. , Hoffmann, A. , Kellermann, V. , Sgrò, C. , McEvey, S. , & Ferrier, S. (2016). Incorporating evolutionary adaptation in species distribution modelling reduces projected vulnerability to climate change. Ecology Letters, 19(12), 1468–1478. 10.1111/ele.12696 27873482

[jane70332-bib-0009] Cereja, R. (2020). Critical thermal maxima in aquatic ectotherms. Ecological Indicators, 119, 106856. 10.1016/j.ecolind.2020.106856

[jane70332-bib-0010] Claussen, D. (1977). Thermal acclimation in ambystomatid salamanders. Comparative Biochemistry and Physiology Part A: Physiology, 58(4), 333–340. 10.1016/0300-9629(77)90150-5

[jane70332-bib-0011] De Bonville, J. , Andreassen, A. H. , Cowan, Z.‐L. , Silva‐Garay, L. , Leeuwis, R. H. J. , Åsheim, E. R. , Speers‐Roesch, B. , Raby, G. D. , Binning, S. A. , & Jutfelt, F. (2025). Dynamics of thermal tolerance plasticity across fish species and life stages. Journal of Thermal Biology, 127, 104024. 10.1016/j.jtherbio.2024.104024 39721158

[jane70332-bib-0012] De Jong, G. (2005). Evolution of phenotypic plasticity: Patterns of plasticity and the emergence of ecotypes. New Phytologist, 166(1), 101–118. 10.1111/j.1469-8137.2005.01322.x 15760355

[jane70332-bib-0013] Donelson, J. M. , Sunday, J. M. , Figueira, W. F. , Gaitán‐Espitia, J. D. , Hobday, A. J. , Johnson, C. R. , Leis, J. M. , Ling, S. D. , Marshall, D. , Pandolfi, J. M. , Pecl, G. , Rodgers, G. G. , Booth, D. J. , & Munday, P. L. (2019). Understanding interactions between plasticity, adaptation and range shifts in response to marine environmental change. Philosophical Transactions of the Royal Society, B: Biological Sciences, 374(1768), 20180186. 10.1098/rstb.2018.0186 PMC6365866 30966966

[jane70332-bib-0063] Duarte, C. M. , Middelburg, J. J. , & Caraco, N. (2005). Major role of marine vegetation on the oceanic carbon cycle. Biogeosciences, 2(1), 1–8. 10.5194/bg-2-1-2005

[jane70332-bib-0014] Einum, S. , Ratikainen, I. , Wright, J. , Pélabon, C. , Bech, C. , Jutfelt, F. , Stawski, C. , & Burton, T. (2019). How to quantify thermal acclimation capacity? Global Change Biology, 25(6), 1893–1894. 10.1111/gcb.14598 30779405

[jane70332-bib-0015] Ern, R. , Andreassen, A. H. , & Jutfelt, F. (2023). Physiological mechanisms of acute upper thermal tolerance in fish. Physiology, 38(3), 141–158. 10.1152/physiol.00027.2022 36787401

[jane70332-bib-0016] GBIF.org User . (2025). Occurrence download . (p. 1467516) [Darwin Core Archive,text/tab‐separated‐values,application/zip]. The Global Biodiversity Information Facility 10.15468/DL.JYZPHR

[jane70332-bib-0017] Gunderson, A. R. (2023). Trade‐offs between baseline thermal tolerance and thermal tolerance plasticity are much less common than it appears. Global Change Biology, 29(13), 3519–3524. 10.1111/gcb.16710 37272873

[jane70332-bib-0018] Gunderson, A. R. , & Stillman, J. H. (2015). Plasticity in thermal tolerance has limited potential to buffer ectotherms from global warming. Proceedings of the Royal Society B: Biological Sciences, 282(1808), 20150401. 10.1098/rspb.2015.0401 PMC4455808 25994676

[jane70332-bib-0019] Hobday, A. J. , Alexander, L. V. , Perkins‐Kirkpatrick, S. E. , Smale, D. A. , Straub, S. C. , Oliver, E. C. J. , Benthuysen, J. A. , Burrows, M. T. , Donat, M. G. , Feng, M. , Holbrook, N. J. , Moore, P. J. , Scannell, H. A. , Sen Gupta, A. , & Wernberg, T. (2016). A hierarchical approach to defining marine heatwaves. Progress in Oceanography, 141, 227–238. 10.1016/j.pocean.2015.12.014

[jane70332-bib-0066] Hudson, J. , Johannesson, K. , McQuaid, C. D. , & Rius, M. (2019). Secondary contacts and genetic admixture shape colonization by an amphiatlantic epibenthic invertebrate. Evolutionary Applications, 13(3), 600–612. 10.1111/eva.12893 PMC7045719 32431738

[jane70332-bib-0020] IPCC . (2023). Climate change 2023: Synthesis report. Contribution of working groups I, II and III to the sixth assessment report of the intergovernmental panel on climate change [Core Writing Team, H. Lee and J. Romero (eds.)]. IPCC. 10.59327/IPCC/AR6-9789291691647

[jane70332-bib-0021] Janzen, D. H. (1967). Why mountain passes are higher in the tropics. The American Naturalist, 101, 233–249.

[jane70332-bib-0022] Jensen, D. L. , Overgaard, J. , Wang, T. , Gesser, H. , & Malte, H. (2017). Temperature effects on aerobic scope and cardiac performance of European perch (*Perca fluviatilis*). Journal of Thermal Biology, 68, 162–169. 10.1016/j.jtherbio.2017.04.006 28797476

[jane70332-bib-0023] Jørgensen, L. B. , Malte, H. , & Overgaard, J. (2019). How to assess *drosophila* heat tolerance: Unifying static and dynamic tolerance assays to predict heat distribution limits. Functional Ecology, 33(4), 629–642. 10.1111/1365-2435.13279

[jane70332-bib-0024] Jutfelt, F. , Ern, R. , Leeuwis, R. H. J. , & Clark, T. D. (2024). Effects of climate warming. In Encyclopedia of fish physiology (pp. 14–31). Elsevier. 10.1016/B978-0-323-90801-6.00183-X

[jane70332-bib-0025] Kuznetsova, A. , Brockhoff, P. B. , & Christensen, R. H. B. (2017). lmerTest package: Tests in linear mixed effects models. Journal of Statistical Software, 82(13), 1–26. 10.18637/jss.v082.i13

[jane70332-bib-0026] Lenth, R. V. , & Piaskowski, J. (2017). *emmeans: Estimated Marginal Means*, *aka Least‐Squares Means* (p. 2.0.0) [Dataset]. 10.32614/CRAN.package.emmeans

[jane70332-bib-0027] Lüdecke, D. , Patil, I. , Ben‐Shachar, M. , Wiernik, B. , Waggoner, P. , & Makowski, D. (2021). See: An R package for visualizing statistical models. The Journal of Open Source Software, 6(64), 3393. 10.21105/joss.03393

[jane70332-bib-0028] MacArthur, R. H. (1984). Geographical ecology: Patterns in the distribution of species. Princeton university press.

[jane70332-bib-0029] Madeira, C. , Mendonça, V. , Leal, M. C. , Diniz, M. S. , Cabral, H. N. , Flores, A. A. V. , & Vinagre, C. (2019). Present and future invasion perspectives of an alien shrimp in South Atlantic coastal waters: An experimental assessment of functional biomarkers and thermal tolerance. Biological Invasions, 21(5), 1567–1584. 10.1007/s10530-019-01921-1

[jane70332-bib-0030] Massicotte, P. , & South, A. (2017). rnaturalearth: World Map Data from Natural Earth . (p. 1.1.0) [Dataset] 10.32614/CRAN.package.rnaturalearth

[jane70332-bib-0031] Morgan, R. , Finnøen, M. H. , Jensen, H. , Pélabon, C. , & Jutfelt, F. (2020). Low potential for evolutionary rescue from climate change in a tropical fish. Proceedings of the National Academy of Sciences of the United States of America, 117(52), 33365–33372. 10.1073/pnas.2011419117 PMC7776906 33318195

[jane70332-bib-0032] Morley, S. A. , Peck, L. S. , Sunday, J. M. , Heiser, S. , & Bates, A. E. (2019). Physiological acclimation and persistence of ectothermic species under extreme heat events. Global Ecology and Biogeography, 28(7), 1018–1037. 10.1111/geb.12911

[jane70332-bib-0033] Noble, D. W. A. , Pottier, P. , Lagisz, M. , Burke, S. , Drobniak, S. M. , O'Dea, R. E. , & Nakagawa, S. (2022). Meta‐analytic approaches and effect sizes to account for ‘nuisance heterogeneity’ in comparative physiology. Journal of Experimental Biology, 225(Suppl_1), jeb243225. 10.1242/jeb.243225 35258606

[jane70332-bib-0034] Oliver, E. C. J. , Donat, M. G. , Burrows, M. T. , Moore, P. J. , Smale, D. A. , Alexander, L. V. , Benthuysen, J. A. , Feng, M. , Sen Gupta, A. , Hobday, A. J. , Holbrook, N. J. , Perkins‐Kirkpatrick, S. E. , Scannell, H. A. , Straub, S. C. , & Wernberg, T. (2018). Longer and more frequent marine heatwaves over the past century. Nature Communications, 9(1), 1324. 10.1038/s41467-018-03732-9 PMC5893591 29636482

[jane70332-bib-0035] Parry‐Wilson, H. M. , Fenberg, P. B. , Hawkins, S. J. , & Mieszkowska, N. (2024). Lethal and sub‐lethal responses of rocky shore gastropods to extreme temperatures. Journal of Experimental Marine Biology and Ecology, 579, 152044. 10.1016/j.jembe.2024.152044

[jane70332-bib-0036] Pebesma, E. (2016). sf: Simple Features for R . (p. 1.0–21) [Dataset] 10.32614/CRAN.package.sf

[jane70332-bib-0037] Peck, L. S. , Morley, S. A. , Richard, J. , & Clark, M. S. (2014). Acclimation and thermal tolerance in Antarctic marine ectotherms. Journal of Experimental Biology, 217(1), 16–22. 10.1242/jeb.089946 24353200

[jane70332-bib-0038] Pinsky, M. L. , Eikeset, A. M. , McCauley, D. J. , Payne, J. L. , & Sunday, J. M. (2019). Greater vulnerability to warming of marine versus terrestrial ectotherms. Nature, 569(7754), 108–111. 10.1038/s41586-019-1132-4 31019302

[jane70332-bib-0065] Posit Team . (2025). RStudio: Integrated development environment for R (Version 2025.05.1+531). Posit Software, PBC. https://posit.co/

[jane70332-bib-0039] Pottier, P. , Burke, S. , Drobniak, S. M. , Lagisz, M. , & Nakagawa, S. (2021). Sexual (in)equality? A meta‐analysis of sex differences in thermal acclimation capacity across ectotherms. Functional Ecology, 35(12), 2663–2678. 10.1111/1365-2435.13899

[jane70332-bib-0040] Pottier, P. , Burke, S. , Zhang, R. Y. , Noble, D. W. A. , Schwanz, L. E. , Drobniak, S. M. , & Nakagawa, S. (2022). Developmental plasticity in thermal tolerance: Ontogenetic variation, persistence, and future directions. Ecology Letters, 25(10), 2245–2268. 10.1111/ele.14083 PMC9804923 36006770

[jane70332-bib-0041] Pottier, P. , Noble, D. W. A. , Seebacher, F. , Wu, N. C. , Burke, S. , Lagisz, M. , Schwanz, L. E. , Drobniak, S. M. , & Nakagawa, S. (2024). New horizons for comparative studies and meta‐analyses. Trends in Ecology & Evolution, 39(5), 435–445. 10.1016/j.tree.2023.12.004 38216408

[jane70332-bib-0064] R Core Team . (2025). R: A language and environment for statistical computing (Version 4.5.1). R Foundation for Statistical Computing. https://www.R‐project.org/

[jane70332-bib-0068] Raby, G. D. , Morgan, R. , Andreassen, A. H. , Stewart, E. M. C. , De Bonville, J. , Hoots, E. C. , Kuchenmüller, L. , Metz, M. , Rowsey, L. E. , Green, L. , Griffin, R. A. , Martin, S. , Reid, H. B. , Ern, R. , Åsheim, E. R. , Cowan, Z.‐L. , Leeuwis, R. H. J. , Blewett, T. A. , Speers‐Roesch, B. , … Jutfelt, F. (2025). Measuring critical thermal maximum in aquatic ectotherms: A practical guide. Methods in Ecology and Evolution, 16(10), 2208–2228. 10.1111/2041-210x.70103

[jane70332-bib-0043] Ravaux, J. , Léger, N. , Rabet, N. , Fourgous, C. , Voland, G. , Zbinden, M. , & Shillito, B. (2016). Plasticity and acquisition of the thermal tolerance (upper thermal limit and heat shock response) in the intertidal species *Palaemon elegans* . Journal of Experimental Marine Biology and Ecology, 484, 39–45. 10.1016/j.jembe.2016.07.003

[jane70332-bib-0044] Rodgers, E. M. , & Gomez Isaza, D. F. (2023). The mechanistic basis and adaptive significance of cross‐tolerance: A ‘pre‐adaptation’ to a changing world? Journal of Experimental Biology, 226(11), jeb245644. 10.1242/jeb.245644 37288646

[jane70332-bib-0045] Rodil, I. F. , Lohrer, A. M. , Attard, K. M. , Thrush, S. F. , & Norkko, A. (2022). Positive contribution of macrofaunal biodiversity to secondary production and seagrass carbon metabolism. Ecology, 103(4), e3648. 10.1002/ecy.3648 PMC9287067 35080770

[jane70332-bib-0067] Sardain, A. , Sardain, E. , & Leung, B. (2019). Global forecasts of shipping traffic and biological invasions to 2050. Nature Sustainability, 2(4), 274–282. 10.1038/s41893-019-0245-y

[jane70332-bib-0046] Seebacher, F. , White, C. R. , & Franklin, C. E. (2015). Physiological plasticity increases resilience of ectothermic animals to climate change. Nature Climate Change, 5(1), 61–66. 10.1038/nclimate2457

[jane70332-bib-0047] Smith, K. E. , Aubin, M. , Burrows, M. T. , Filbee‐Dexter, K. , Hobday, A. J. , Holbrook, N. J. , King, N. G. , Moore, P. J. , Sen Gupta, A. , Thomsen, M. , Wernberg, T. , Wilson, E. , & Smale, D. A. (2024). Global impacts of marine heatwaves on coastal foundation species. Nature Communications, 15(1), 5052. 10.1038/s41467-024-49307-9 PMC11176324 38871692

[jane70332-bib-0048] Smith, K. E. , Burrows, M. T. , Hobday, A. J. , King, N. G. , Moore, P. J. , Sen Gupta, A. , Thomsen, M. S. , Wernberg, T. , & Smale, D. A. (2023). Biological impacts of marine heatwaves. Annual Review of Marine Science, 15(1), 119–145. 10.1146/annurev-marine-032122-121437 35977411

[jane70332-bib-0049] Somero, G. N. (2010). The physiology of climate change: How potentials for acclimatization and genetic adaptation will determine ‘winners’ and ‘losers’. Journal of Experimental Biology, 213(6), 912–920. 10.1242/jeb.037473 20190116

[jane70332-bib-0050] Stillman, J. H. (2003). Acclimation capacity underlies susceptibility to climate change. Science, 301(5629), 65. 10.1126/science.1083073 12843385

[jane70332-bib-0051] Stillman, J. H. , & Somero, G. N. (2000). A comparative analysis of the upper thermal tolerance limits of eastern Pacific porcelain crabs, genus *Petrolisthes*: Influences of latitude, vertical zonation, acclimation, and phylogeny. Physiological and Biochemical Zoology, 73(2), 200–208. 10.1086/316738 10801398

[jane70332-bib-0052] Sunday, J. , Bennett, J. M. , Calosi, P. , Clusella‐Trullas, S. , Gravel, S. , Hargreaves, A. L. , Leiva, F. P. , Verberk, W. C. E. P. , Olalla‐Tárraga, M. Á. , & Morales‐Castilla, I. (2019). Thermal tolerance patterns across latitude and elevation. Philosophical Transactions of the Royal Society, B: Biological Sciences, 374(1778), 20190036. 10.1098/rstb.2019.0036 PMC6606462 31203755

[jane70332-bib-0053] Tamarit Castro, E. , Steinbrecher, F. , Pfeufer, L. , Lechner, E. R. , Sauer, H. , Green, L. , Perry, D. , Linderholm, H. , Gullstrom, M. , & Jutfelt, F. (2026). Fishes in eelgrass meadows show large interspecific differences in thermal acclimation to marine heatwaves. Global Change Biology Communications, 2, e70027. 10.1002/gcb4.70027

[jane70332-bib-0054] Tomás, M. , Pottier, P. , Alexandro, A. , Tamarit‐Castro, E. , Alexander, B. , Blewett, T. , Ivan, C. , Doty, D. , Gradito, M. , Hoots, E. , Kuchenmüller, L. , Martin, S. , Mélançon, V. , Vigneault, J. , Clark, T. , Cowan, Z.‐L. , Jutfelt, F. , & Green, L. (2025). *Limited capacity for rapid acclimation to marine heatwaves in coastal invertebrates* [Dataset]. Zenodo 10.5281/zenodo.19691547 PMC13643194 42605131

[jane70332-bib-0056] Tyberghein, L. , Verbruggen, H. , Pauly, K. , Troupin, C. , Mineur, F. , & De Clerck, O. (2012). Bio‐ORACLE: A global environmental dataset for marine species distribution modelling. Global Ecology and Biogeography, 21(2), 272–281. 10.1111/j.1466-8238.2011.00656.x

[jane70332-bib-0057] Van Heerwaarden, B. , & Kellermann, V. (2020). Does plasticity trade off with basal heat tolerance? Trends in Ecology & Evolution, 35(10), 874–885. 10.1016/j.tree.2020.05.006 32513551

[jane70332-bib-0058] Viechtbauer, W. (2010). Conducting meta‐analyses in *R* with the metafor package. Journal of Statistical Software, 36(3), 1–48. 10.18637/jss.v036.i03

[jane70332-bib-0059] Vinagre, C. , Dias, M. , Cereja, R. , Abreu‐Afonso, F. , Flores, A. A. V. , & Mendonça, V. (2019). Upper thermal limits and warming safety margins of coastal marine species – Indicator baseline for future reference. Ecological Indicators, 102, 644–649. 10.1016/j.ecolind.2019.03.030

[jane70332-bib-0060] Vinagre, C. , Leal, I. , Mendonça, V. , Madeira, D. , Narciso, L. , Diniz, M. S. , & Flores, A. A. V. (2016). Vulnerability to climate warming and acclimation capacity of tropical and temperate coastal organisms. Ecological Indicators, 62, 317–327. 10.1016/j.ecolind.2015.11.010

[jane70332-bib-0061] Wernberg, T. , Thomsen, M. S. , Baum, J. K. , Bishop, M. J. , Bruno, J. F. , Coleman, M. A. , Filbee‐Dexter, K. , Gagnon, K. , He, Q. , Murdiyarso, D. , Rogers, K. , Silliman, B. R. , Smale, D. A. , Starko, S. , & Vanderklift, M. A. (2024). Impacts of climate change on marine foundation species. Annual Review of Marine Science, 16(1), 247–282. 10.1146/annurev-marine-042023-093037 37683273

[jane70332-bib-0062] Willmer, P. , Stone, G. , & Johnston, I. A. (2005). Environmental physiology of animals (2nd ed.) Blackwell Publications.

