# Supplementary material for: Limited capacity for rapid acclimation to marine heatwaves in coastal invertebrates
Source: J Anim Ecol. 2026 Aug 16;95(10):2088–103. doi: 10.1111/1365-2656.70332 (PMC13643194; doi:10.1111/1365-2656.70332)
Supplement: Supplementary file 1 [file JANE-95-2088-s001.docx]

**Supplementary Material:**

**Limited capacity for rapid acclimation to marine heatwaves in coastal invertebrates**

Supplementary tables

***Table S1. Experimental design and acclimation conditions for intertidal species exposed to 15°C and 20°C temperature treatments.*** The table provides the collection date or collection period, initial holding temperature, species, treatment temperature, replicate tank number, sample size, acclimation start date and time, temperature measurements during the ramping phase, daily tank temperatures throughout the acclimation period, sampling date and sampling time, tank dimensions, and tank volume. Temperature ramping values indicate the measured tank temperatures at successive time points after the start of acclimation, followed by daily temperature recordings from Day 2 to Day 5. Tank dimensions are reported as length × width × height, and tank volume is reported in litres. The table documents the acclimation history and sampling details for each replicate tank prior to experimental measurements. The complete table is also available in Excel format together with the associated data.

***Table S2. Sensitivity analysis of thermal tolerance (CT_max_) based on linear mixed-effects models that include all individuals, including those that did not recover 30 minutes post-trial.*** The first model tested the effects of treatment and its interaction with species, focusing on whether treatment responses differed between species. The second model additionally included species, body mass, and their interactions to assess species-specific mass effects. R²_m_ represents the marginal R², while R²_c_ represents the conditional R². Significant predictors (*p* < 0.05) are shown in bold. Denominator degrees of freedom were estimated using Satterthwaite’s method.

***Table S3. Model comparison results for the main and sensitivity linear mixed-effects models of thermal tolerance (CT_max_), generated using the performance package.*** The table summarizes differences in model fit metrics between the primary model (including only individuals that recovered) and the sensitivity model (including both recovered and non-recovered individuals). Reported statistics include Akaike Information Criterion (AIC), AIC corrected for small sample sizes (AICc), Bayesian Information Criterion (BIC), marginal and conditional R² (R²_m_ and R²_c_), intraclass correlation coefficient (ICC), root mean square error (RMSE), and residual standard deviation (Sigma). AIC weights reflect the relative support for each model. Lower AIC/BIC values and higher R² values indicate better model performance.

***Table S4. Phylogenetic signal in main responses.*** Phylogenetic signal was assessed for three species-level thermal traits: mean critical thermal maximum (CT_max_), acclimation response ratio (ARR), and mean thermal safety margin (TSM). Species relationships were based on an Open Tree of Life induced subtree, with branch lengths assigned using Grafen’s method. Blomberg’s K was used to test for phylogenetic signal, with significance assessed by randomisation tests. Pagel’s lambda was also estimated to quantify phylogenetic covariance, with significance tested against lambda = 0 using likelihood-ratio tests. *n* indicates the number of species included in each analysis.

***Table S5. Main thermal trait models with and without phylogenetic correction.*** Statistical models for CT_max_, TSM, and ARR were compared with and without phylogenetic correction. For CT_max_ and TSM, mixed models included acclimation treatment as a fixed effect, with tank and species identity as random effects; phylogenetic models additionally included a phylogenetic species-level random effect based on the Open Tree of Life phylogeny. Treatment was coded as 0 for 15°C and 1 for 20°C, so the intercept represents the estimated value at 15°C and the slope represents the difference between 20°C and 15°C treatments. For ARR, intercept-only species-level models were used to estimate the mean acclimation response across species, comparing weighted linear models with Brownian-motion PGLS models. Sample sizes, estimates, standard errors are reported for model comparison. Results should be interpreted cautiously because analyses were based on 10 species

***Table S6. Species-level predictor models with and without phylogenetic correction.*** Species-level associations responses and geographic or environmental predictors were compared using non-phylogenetic linear models and Brownian-motion phylogenetic generalized least squares models. Separate models were fitted for each thermal response variable, Ctmax and ARR and for each predictor. Predictors were mean-centred, so intercepts represent expected trait values at the average predictor value, while slopes represent the change in the response per one-unit increase in the predictor. ARR models were weighted by inverse sampling variance. Estimates, standard errorsand the number of species are reported for each model. Results should be interpreted cautiously because analyses were based on 10 species.

**Table S7. *Summary of thermal tolerance metrics CT_max_ and acclimation response ratio (ARR) for each species included in the experiment.*** For each species, the table shows the taxonomic phylum, sample size (*N*), treatment group, mean CT_max_ (°C) ± standard error (SE), mean ARR ± SE, the difference in CT_max_ between treatment groups (ΔCT_max_) and *p*-value.

***Table S8. Summary of the geographical distribution of selected marine species based on occurrence records from the GBIF database.*** For all species except *H. takanoi*, only European records were considered; for *H. takanoi*, its natural (native) range was used due to its status as an invasive species in Europe. The table shows the mean, minimum, and maximum recorded latitudes (in decimal degrees), the resulting latitudinal range (maximum − minimum latitude), and the total number of occurrence records (N) used in the analysis.

***Table S9. Results of linear mixed-effects models and meta-analytic mixed-effects models testing the effects of SST metrics on CT_max_ and ARR, respectively.*** For CT_max,_ models included acclimation treatment (15 °C control vs. heatwave) and a single SST as fixed effects. Denominator degrees of freedom (df) were estimated using Satterthwaite’s approximation. For ARR, separate random-effects meta-regressions were fitted using species as the grouping factor and each SST metric as a moderator. For CT_max_ models, R²ₘ represents the variance explained by fixed effects and R²_c_ the variance explained by fixed plus random effects. For ARR models, R² represents the proportion of between-study heterogeneity explained by the moderator.

***Table S10. Thermal safety margins (TSM) across species and acclimation treatments.*** Values are reported as mean TSM (°C) ± SD, with standard error (SE) and sample size (N, number of individuals) for each species and treatment. TSM was calculateed as TSM = CT_max_ − T_max,env,_ where CT_max_ is the experimentally determined critical thermal maximum (°C) and T_max,env_ is the local maximum sea surface temperature (°C) derived from Bio-ORACLE.

Supplementary figures

**Figure S1. Seasonal and short-term thermal variability at 1 m depth in Gullmarsfjorden from 2020–2025.** **A)** Daily sea temperature patterns, showing the 30-day rolling mean temperature in red and the observed daily minimum–maximum temperature range in pale red. Dashed horizontal lines indicate the overall mean minimum and mean maximum temperatures across the study period. **B)** Daily thermal variation, calculated as the difference between daily maximum and minimum temperature. Pale red bars show observed daily ranges, while the red line shows the 30-day rolling mean daily range**. C)** Seasonal temperature structure comparing winter and summer mean temperatures across years. Thin red curves show individual yearly seasonal curves, the thicker red curve shows the mean seasonal curve, and the pale red band indicates the minimum–maximum range across years. The mean summer–winter temperature difference was **13.18 °C.**

***Figure S2. Phylogenetic relationships among the study species.*** Phylogenetic tree showing the relationships among the 10 invertebrate species used in analyses of CTmax, ARR, and TSM. The tree was generated from an Open Tree of Life induced subtree using species-level taxonomic matches, with branch lengths assigned using Grafen’s method. This phylogeny was used to assess phylogenetic signal and to fit phylogenetically corrected comparative models. Species names are shown at the tips, and the tree represents the hypothesised evolutionary relationships among the sampled species.

***Figure S3. Mean sea surface temperature (SST; °C) across European waters from 2010 to 2020, overlaid with occurrence records for ten marine invertebrate species.*** The background represents decadal mean SST data obtained from Bio-ORACLE, while occurrence points were compiled from the Global Biodiversity Information Facility (GBIF). Each panel shows the spatial distribution of one species, with the number of occurrence records shown in parentheses.

***Figure S4. Effect of body mass on CT_max_ across two treatment groups (control and heatwave +5 °C) for all species included in the experiment.*** Each panel shows individual CT_max_ measurements (points) plotted against body mass for a single species. Red points represent individuals from the heatwave treatment, and blue points represent individuals from the control treatment.

***Figure S5.*** ***Spatial summary of sea surface temperature (SST, °C) across European waters for the period 2010–2020.*** Each panel depicts a different decadal summary statistic derived from daily SST data: **(a)** long-term maximum, **(b)** long-term minimum, **(c)** instantaneous maximum, **(d)** mean, **(e)** instantaneous minimum, and **(f)** range (difference between long-term maximum and minimum). Warmer colours represent higher temperatures and cooler colours represent lower temperatures.

***Figure S6. Relationships between CT_max_ (panels A–F) and ARR (panels G–L) and six sea surface temperature (SST) metrics across species.*** For CT_max_ (A–F), points represent individual measurements from two acclimation treatments: blue = 15 °C control, red = heatwave treatment. Lines show model predictions from linear mixed-effects models with treatment and one SST variable as fixed effects and species as a random intercept; shaded ribbons denote 95% confidence intervals. Panels G–L show relationships between ARR and the same SST metrics based on random-effects meta-regressions. Each point represents a study-level effect size, with circle area proportional to precision (1/SE). Red lines indicate model predictions with 95% confidence ribbons.

Extended Methods

**Animal collection and husbandry**

We collected individuals on multiple occasions in June 2025 from Bökevik Bay, adjacent to the Kristineberg Marine Research Station, Sweden (58°'24.9"N 11°44'58.5"E). All animal collection and experimental work took place between June 20 and July 5, 2025. We captured crustaceans (*C. crangon*, *P. flexuosus*, *C. maenas*, and *P. elegans*) using a beach seine (1 × 8 m, 3 mm mesh) in shallow sandy areas (0–2 m depth). We hand-netted *A. papillosa*, *H. takanoi*, *L. littorea*, *A. rubens*, *P. bernhardus*, and *T. nitida* while snorkelling in the same bay. We also obtained additional *A. rubens* from baited cages suspended from the station’s docks (1–3 m depth).

After capture, we maintained the animals in flow-through glass aquaria (85 × 26 × 35 cm [L × W × H]; approximately 77 L) that received fjord seawater drawn from 7 m depth, maintaining conditions comparable to the collection site (14.9 °C ± 0.3 °C; salinity 29.20 ± 0.01 PSU; means ± standard deviation (SD)), and set the photoperiod to 18 h light: 6 h dark to match local conditions. To prevent aggressive interactions and predation, we roughly size-sorted *C. maenas*, *H. takanoi*, and *P. bernhardus*, then housed them separately in species-specific aquaria (60 × 38 × 36 cm [L × W × H]; approximately 30 L). Each aquarium contained plastic algae for enrichment, and we fed the animals daily with brine shrimp (*Artemia salina*; Ocean Nutrition). Collection dates and individual holding durations appear in Supplementary Table 1.

**Acclimation treatments**

We conducted acclimation treatments in a temperature-controlled room at the Kristineberg Marine Research Station, which received continuous flow-through seawater (salinity 29.20 ± 0.01 PSU) drawn from a depth of 7 m. We transferred individuals from holding aquaria into food-grade, semi-transparent plastic containers (36 × 26.5 × 17 cm [L × W × H]; approximately 12.20 L, or 27 × 17 × 12.5 cm [L × W × H], 5.30 L; the three crab species were placed in the larger containers to minimise aggressive interactions).

We exposed the animals to one of two temperature regimes: a control treatment (15.28 ± 0.26 °C) representing ambient field conditions, or a heatwave treatment (20.14 ± 0.21 °C), corresponding to an increase of about 5 °C above ambient. A +5 °C anomaly is consistent with Severe-to-Extreme marine heatwave conditions in the Skagerrak region under the Hobday et al. (2018) classification framework, and comparable magnitudes have been recorded during recent events in adjacent waters (Filbee-Dexter et al., 2020). A recent study from the same region used a comparable +4 °C treatment validated as representative of current summer conditions in local eelgrass habitats (Tamarit Castro et al., 2026). The five-day exposure also meets the minimum duration criterion in the Hobday et al. (2016) definition of a marine heatwave. For each species, we assigned three replicate containers to each treatment, stocking ranging from 10 to 13 individuals per experimental tank. To prevent theraml shock, the targeted heatwave temperature was achieved by gradually mixing heated and control inflows over a 24-h period. The fraction of warm water was adjusted every few hours, allowing the system to gradually reach the target heatwave temperature (Supplementary Table 1). After this ramping phase, we held animals at their treatment temperatures for four consecutive days to simulate a short heatwave. Animals were not directly fed during the acclimation period; however, flow-through seawater provided a continuous supply of natural particulate material, which may have served as a nutritional resource.

We checked water flow, animal condition, and mortality daily during acclimation. We removed dead individuals immediately (N=11; *C. maenas*, *C. crangon* and *A. rubens*), but left moults and deposited egg masses (from the nudibranch *A. papillosa*) in place. We monitored temperature in each container daily using digital thermometers (Testo 112), calibrated against a laboratory-grade reference thermometer (Testo 735-1, serial #60810957). We maintained dim lighting with overhead fluorescent bulbs to limit algal growth, using a 18 h light: 6 h dark photoperiod, with a simulated dawn (50% light from 06:00 to 08:00) and dusk (50% light from 22:00 to 23:00) to mimic natural cycles. Supplementary Table 1 provides heating profiles and daily temperature records.

**Upper thermal tolerance trials**

After the acclimation period, we determined CT_max_ following a standardised protocol adapted from [Raby et al., (2025)](https://www.zotero.org/google-docs/?wzn4bE). We transferred individuals to a 12 L tank (25 × 20 × 18 cm [L × W × H]) partitioned with mesh to separate the animal arena from the heating compartment, which contained a 300 W coil heater and a circulation pump (5 L min⁻¹; Eheim Universal 300, Germany). We filled the tank with water at the individual's acclimation temperature and continuously aerated using a pump (60 L h⁻¹, Marina 75 pump) fitted with an air stone to maintain oxygen saturation. Starting CTmax trials at each group's acclimation temperature is standard practice for multi-acclimation comparisons (Terblanche et al., 2007; Terblanche & Hoffmann, 2020). Bringing all groups to a common intermediate temperature would require an abrupt thermal shift immediately before the assay, which could itself trigger a stress response and confound the comparison. We allowed individuals to habituate for 10 min before heating began. We increased the temperature at a rate of approximately 0.3 °C min⁻¹ (mean ± SD: 0.303 ± 0.007 °C min⁻¹). We closely monitored the heating using a Testo 112 thermometer (accuracy ± 0.2 °C, resolution 0.1 °C) calibrated against a laboratory reference (Testo 735-1, serial #60810957).

Observers conducting the trials were blinded to treatment temperature for all trials, except for two nudibranch trials where blinding was not maintained at the start. We mitigated potential bias by including at least one experienced observer per trial and using pre-defined, species-specific criteria for scoring loss of motor function (Table 1), aiming to standardize endpoint assessment as objectively as possible. Behavioural responses varied among species but generally included loss of grip and inability to stay attached to the substrate, or increased movement followed by immobility and lack of response to tactile stimulation by the observer. Although these differ in overt expression they share a common physiological interpretation: the organism has lost the capcity for direct, coordinated movement. We assessed responsiveness by gently applying a soft brush to minimise handling stress. However, we adjusted the criteria for two species due to their behavioural traits. *A. papillosa* remained largely immobile during CT_max_ trials and showed little agitation. Pilot trials showed that individuals could right themselves quickly after being flipped, so after prolonged stillness (over 1 min), we gently turned individuals upside down; inability to right themselves indicated loss of motor function. By contrast, *T. nitida* exhibited high mobility under normal conditions, with individuals frequently flipping. At higher temperatures, individuals became unusually still but did not detach or display visible colour changes. As righting behaviour is normally fast, loss of motor function was scored when individuals were unable to righ themselves or required longer than 30 second to do so after being inverted. We also recorded absence of siphon and core retraction upon gentle tactile stimulation as a secondary endpoint, confirming cessation of motor responses.

We ran two to three trials in parallel, staggering start times by 10–15 min to allow careful monitoring of all individuals. To standardise procedures and minimise temporal bias, we fully randomised trial order across acclimation treatments and replicates within each species, resulting in an interspersed sequence of warm and control-acclimated individuals throughout the experiment. Once we observed loss of motor function, we immediately transferred the animal to an individual recovery tank (16 ± 1 °C in the control treatment and 20 ± 1 °C in the heatwave treatment) and observed it for 30 min. After this period, we assessed recovery (regaining movement), recorded mortality, and weighed individuals to the nearest 0.001 g (ABT 124-AM, Kern) after removing excess water (metal sieve for most species; gentle blotting with absorbent tissue paper for sea stars due to their larger size). We returned all individuals to Bökevik Bay after completing the study.

**Statistical analyses**

We performed all statistical analyses and data visualisation using R (version 4.5.1; R Core Team, 2025) through RStudio (version 2025.05.1+513; RStudio Team, 2025). For our main analyses, we included only individuals who survived the experimental procedures. However, we re-ran all analyses to include individuals who did not survive to check for potential bias, and we present the results from this sensitivity analysis in the Supplementary Tables 2 and 3. We observed high overall survival (98.3%), so we considered the few mortalities (N=10) to reflect abnormal responses and excluded them as outliers. Furthermore, we excluded the first trial with *P. bernhardus* because the behavioural responses for loss of motor function did not meet the criteria set in the pilot studies (see Supplementary Table 1 for details). For all models, we set statistical significance at *p* < 0.05.

**2.4.1. Variation in thermal tolerance**

We analysed intra and interspecific variation in thermal tolerance by fitting a single linear mixed-effects model (LMM) with the lmer function (lme4; [Bates et al., 2015)](https://www.zotero.org/google-docs/?kSTu5c). In our baseline model, we specified treatment (control vs. heatwave), species, and their interaction as fixed effects, and set tank identity as a random intercept to account for potential pseudoreplication from shared rearing environments. To evaluate if body mass influenced variation in thermal tolerance, we scaled individual mass to a mean of zero and variance of one and included it as a covariate in additional models. For each species, we fit models both with and without mass and inspected residuals to confirm model adequacy.

We performed model inference using the lmerTest package [(Kuznetsova et al., 2017)](https://www.zotero.org/google-docs/?OrhROP), which employs Satterthwaite’s approximation for degrees of freedom. To verify the model assumptions of normality and homoscedasticity, we used the performance package [(Lüdecke et al., 2021)](https://www.zotero.org/google-docs/?gzl24a) to check residual diagnostics and found no significant violations. In our complete model, we included treatment, species, and body mass as fixed effects and added a treatment × species interaction to test for species-specific acclimation responses. After testing, we removed body mass interactions that were not significant (*p* > 0.05) for parsimony and clarity. To obtain estimated marginal means (EMMs) and pairwise contrasts, we used the emmeans package [(Lenth & Piaskowski, 2017)](https://www.zotero.org/google-docs/?ypIQqZ), and we adjusted multiple comparisons using Holm or Tukey corrections to control the family-wise error rate.

**2.4.2. Acclimation capacity**

For each species, we quantified acclimation capacity by calculating the change in critical thermal maximum, CT_max_, between acclimation treatments, and expressed it as the ARR following [Claussen (1977)](https://www.zotero.org/google-docs/?v9SwID). We defined ARR as the slope of the relationship between thermal tolerance and acclimation temperature, with:

$\boldsymbol{ARR =}\frac{\boldsymbol{\Delta CTmax}}{\boldsymbol{\Delta T}}\boldsymbol{=}\frac{\boldsymbol{CTmax (20 ^{\circ}C)- CTmax (15 ^{\circ}C)}}{\boldsymbol{20-15}}$	[1],

where CT_max_ (20 °C) and CT_max_ (15 °C) are the mean critical thermal maxima for individuals acclimated to the heatwave (20 °C) and control (15 °C) treatments, respectively, and T is the 5 °C difference in acclimation temperature. Biologically, an ARR value of 1 would indicate perfect thermal compensation, meaning that thermal tolerance increases by 1 °C for every 1 °C increase in acclimation temperature. In contrast, an ARR of 0 indicates no capacity for thermal acclimation [(Pottier et al., 2021)](https://www.zotero.org/google-docs/?yZtwUD).

To determine the precision of ARR estimates, we calculated its sampling variance (ARR2) using the error-propagation method, which incorporates the independent variances of the two means [(Noble et al., 2022; Pottier et al., 2021, 2024)](https://www.zotero.org/google-docs/?HUwQUX). Because ARR is a ratio involving the difference between two independent CT_max_ values, we summed the variances of those values and scaled the sum by the square of the denominator (T2) [(Noble et al., 2022)](https://www.zotero.org/google-docs/?xz6Hzx):

$\sigma_{\boldsymbol{ARR}}^{\boldsymbol{2}}\boldsymbol{=}\frac{\boldsymbol{1}}{\boldsymbol{(\Delta T)}^{\boldsymbol{2}}}\boldsymbol{(}\boldsymbol{SE}_{\boldsymbol{CTmax(20)}}^{\boldsymbol{2}}\boldsymbol{+}\boldsymbol{SE}_{\boldsymbol{CTmax(15)}}^{\boldsymbol{2}}\boldsymbol{)}$	[2],

where T is the 5 °C temperature difference. The final standard error of the ARR used in subsequent analysis was calculated as the square root of this variance.

$\boldsymbol{SE}_{\boldsymbol{ARR}} \frac{\boldsymbol{1}}{\boldsymbol{\Delta T}}\sqrt{\boldsymbol{SE}_{\boldsymbol{CTmax(20)}}^{\boldsymbol{2}}\boldsymbol{+}\boldsymbol{SE}_{\boldsymbol{CTmax(15)}}^{\boldsymbol{2}}}$              [3],

we compared species-level acclimation capacity using weighted meta-analytical models that we implemented in the R package metafor [(Pottier et al., 2024; Viechtbauer, 2010)](https://www.zotero.org/google-docs/?AjGGMu). We weighted each species’ ARR estimate by the inverse of its sampling variance [(Noble et al., 2022)](https://www.zotero.org/google-docs/?ry8iN1). This procedure ensured species with more precise ARR estimates (lower sampling variance, typically from larger sample sizes or lower individual variation) contributed proportionally more to the analysis, while species with greater uncertainty contributed proportionally less [(Pottier et al., 2024)](https://www.zotero.org/google-docs/?PFWTU9).

To complement ARR as a measure of acclimation capacity, we also quantified the short-term change in thermal tolerance for each species, referred to here as the thermal tolerance gain (TT_gain_). TT_gain_ reflects the increase in an organism’s upper thermal limit relative to its baseline (control) condition following exposure to an acclimation treatment [(De Bonville et al., 2025; Fernandes et al., 2023)](https://www.zotero.org/google-docs/?ndswtf). It was calculated as:

${TT}_{gain}= {CT}_{max} (20 ^{\circ}C) -{CT}_{max} (15 ^{\circ}C)$	[4],

where CT_max_ (20 °C) and CT_max_ (15 °C) are the mean critical thermal maxima for individuals acclimated to the heatwave (20 °C) and control (15 °C) treatments, respectively.

**2.4.3. Species distribution data**

We obtained species distribution data from the Global Biodiversity Information Facility ([GBIF.org, 2025](https://www.zotero.org/google-docs/?b4Q0Rt), see Supplementary Table 5, Figure 1). We screened all records to retain only entries with valid geographic coordinates and standardised taxonomic names. To maximise data coverage, we included potential typographical errors, spelling variants, and records with or without year identifiers during taxonomic matching. We harmonised all synonyms and misspellings to align with the currently accepted nomenclature. Except for the invasive crab *H. takanoi*, we restricted distributions to the European range; for *H. takanoi*, we used the global range to more accurately represent the species thermal niche. We retained only records with an occurrence status of “present.” We calculated geographic range descriptors, minimum latitude, maximum latitude, and latitudinal breadth, using geospatial tools in R (packages sf; [Pebesma, 2016](https://www.zotero.org/google-docs/?PGGYbw); and rnaturalearth; [Massicotte & South, 2017](https://www.zotero.org/google-docs/?OeAFeZ)). We defined latitudinal breadth (range extent) as the absolute difference between each species’ maximum and minimum recorded latitude.

We examined correlations between thermal tolerance, acclimation capacity, and species geographic position using two complementary approaches. First, we analysed the relationship between latitude and CT_max_ by fitting separate linear mixed-effects models. In these LMNs individual CT_max_ values grouped by acclimation treatment (15 °C control and 20 °C heatwave), served as the response variable. For each model, we included one geographic metric,  minimum latitude, maximum latitude, or latitudinal breadth, as a fixed effect, and specified species identity and tank ID as random intercepts to control for species-level variation and potential tank-level dependencies.

This formulation accounted for non-independence among individuals within species while we tested for general latitudinal effects on thermal tolerance. We based model inference on Satterthwaite’s approximation for degrees of freedom (lmerTest; [Kuznetsova et al., 2017](https://www.zotero.org/google-docs/?qi3YCi)) and verified assumptions using residual diagnostics (performance; [Lüdecke et al., 2021)](https://www.zotero.org/google-docs/?B7eKHO). Second, we assessed geographic variation in acclimation response ratios (ARR) using meta-regression analyses implemented in the metafor package [(Viechtbauer, 2010)](https://www.zotero.org/google-docs/?QWTwO5). In these models, we incorporated species-level ARR estimates and their sampling variances as effect sizes, while including latitude descriptors (minimum latitude, maximum latitude, or latitudinal range) as moderator variables.

**2.4.4. Sea surface temperature data**

We assessed correlations between sea surface temperature (SST) and thermal tolerance using Bio-ORACLE environmental data layers (version 2.2) spanning 2010–2020 [(Assis et al., 2024; Tyberghein et al., 2012)](https://www.zotero.org/google-docs/?2LxnHp). Bio-ORACLE provides standardised global marine environmental variables at a spatial resolution of 0.05°, derived from daily satellite measurements and summarised into long-term climatologies. For each species’ occurrence location, obtained from GBIF, we extracted six SST descriptors to capture different dimensions of local thermal regimes. We included the long-term maximum, representing the average yearly maximum temperature across the decade, and the long-term minimum, representing the average annual minimum temperature. We also used the instantaneous maximum and instantaneous minimum, which represent the single highest and lowest temperatures recorded at each location during the period, respectively. We defined the mean as the overall decadal average, while the range reflects the typical annual thermal variability, calculated as the average yearly difference between maximum and minimum SST values.

We fitted linear mixed-effects models with CT_max_ as the response variable; treatment (15 °C vs. 20 °C) and one SST descriptor (mean, long-term maximum, long-term minimum, instantaneous maximum, instantaneous minimum, or range) as fixed effects, and species identity and tank ID as random intercepts. This structure accounted for interspecific variation and potential non-independence among individuals from the same tank. We relied on Satterthwaite’s approximation for degrees of freedom (lmerTest; [Kuznetsova et al., 2017)](https://www.zotero.org/google-docs/?MDEIcl) for inference and evaluated model assumptions with residual diagnostics (performance; [Lüdecke et al., 2021)](https://www.zotero.org/google-docs/?TSIbMg).

We analysed ARR values separately using meta-regression models (metafor; [Viechtbauer, 2010)](https://www.zotero.org/google-docs/?OzAGVH). We treated species-level ARR estimates and their sampling variances as effect sizes and included SST descriptors as moderators to test for geographic structuring of acclimation capacity across thermal environments.

In addition to model analyses, we used the same Bio-ORACLE dataset to estimate thermal safety margins (TSM) for each individual under the different acclimation treatments. TSM quantifies the distance between an organism’s upper thermal tolerance limit and the highest environmental temperature it typically experiences, providing an index of susceptibility to thermal stress. We defined thermal safety margin as:

$\boldsymbol{TSM=}\boldsymbol{CT}_{\boldsymbol{max}}\boldsymbol{-}\boldsymbol{T}_{\boldsymbol{max,env}}$	[5],

where  CT_max_ is the individual’s critical thermal maximum (°C) determined experimentally, and T_max,env_ is the local maximum sea surface temperature (°C) derived from Bio-ORACLE’s instantaneous maximum SST layer. Higher TSM values indicate greater thermal buffer capacity, while narrower margins suggest increased vulnerability to warming.

To assess the reliability and environmental relevance of these estimates, we compared Bio-ORACLE-derived temperature data against *in situ* observations from temperature loggers operated by the Swedish Meteorological and Hydrological Institute (SMHI) and the Kristineberg Marine Research Station. These *in situ* datasets provided monthly and seasonal temperature records from coastal monitoring stations near our study region, allowing validation of the satellite-based climatologies and ensuring that local fine-scale variability was adequately represented when interpreting species’ thermal limits and safety margins.
